# Migration ecology of western gray catbirds

**DOI:** 10.1186/s40462-021-00249-7

**Published:** 2021-03-17

**Authors:** Kristen A. Mancuso, Megan A. Fylling, Christine A. Bishop, Karen E. Hodges, Michael B. Lancaster, Katharine R. Stone

**Affiliations:** 1 Department of Biology, University of British Columbia Okanagan, Kelowna, British Columbia Canada; 2 Division of Biological Sciences, University of Montana, Missoula, MT USA; 3 Science and Technology Branch, Environment and Climate Change Canada, Delta, British Columbia Canada; 4 Oliver, Canada; 5 MPG Ranch, Florence, MT USA

**Keywords:** *Dumetella carolinensis*, Full annual cycle, Migration, Migratory connectivity, GPS tracking

## Abstract

**Background:**

For many songbirds in North America, we lack movement details about the full annual cycle, notably outside the breeding season. Understanding how populations are linked spatially between breeding and overwintering periods (migratory connectivity) is crucial to songbird conservation and management. We assessed migratory connectivity for 2 breeding populations of Gray Catbirds (*Dumetella carolinensis*) west of and within the Rocky Mountains by determining migration routes, stopover sites, and overwintering locations. Additionally, we compared apparent annual survivorship for both populations.

**Methods:**

We deployed 39 archival light-level geolocators and 21 Global Positioning System (GPS) tags on catbirds in the South Okanagan Valley, British Columbia, Canada, and 32 geolocators and 52 GPS tags in the Bitterroot River Valley, Montana, USA. These devices allowed us to determine migration routes, stopover sites, overwintering locations, and migratory connectivity. Migratory connectivity was quantified using Mantel’s correlation. We used mark-recapture of colour banded catbirds in both sites to estimate apparent annual survivorship.

**Results:**

We retrieved 6 geolocators and 19 GPS tags with usable data. Gray Catbirds from both populations passed through the Rocky Mountains eastward before heading south towards their overwintering locations in northeastern Mexico and Texas. Stopover sites during fall migration occurred primarily in Montana, Kansas, Oklahoma, and Arkansas. Overwintering locations spanned Texas and 5 states in northeastern Mexico. Individual catbirds used up to 4 distinct sites during the overwintering period. Catbirds separated by almost 500 km during the breeding season overlapped during the non-breeding season, suggesting weak migratory connectivity among western populations (Mantel’s correlation = 0.013, *P*-value = 0.41). Catbird apparent annual survivorship estimates were higher in British Columbia (0.61 ± 0.06 females; 0.64 ± 0.05 males) than in Montana (0.34 ± 0.05 females; 0.43 ± 0.04 males), though the main driver of these differences remain unclear.

**Conclusions:**

Our results provide high precision geographic details during the breeding, migration, and overwintering phases of the annual cycle for western Gray Catbirds. Notably, we found that western catbirds followed the Central Flyway as opposed to the Pacific Flyway. We document that catbirds used multiple sites over winter, contrary to the popular belief that this phase of the annual cycle is stationary for most songbirds.

**Supplementary Information:**

The online version contains supplementary material available at 10.1186/s40462-021-00249-7.

## Background

The decline of North American songbirds is a conservation concern in the Anthropocene [1, 2]. There are major knowledge gaps in the ecology of most migratory bird species, with deficits evident especially during migration and winter because most research is focused on breeding birds [2–5]. The link through space and time between breeding and overwintering periods is important to address, in part because the effects in one season can influence events in another season, such as habitat quality influencing body condition and migration timing [5, 6]. The full annual cycle of migrant songbirds generally includes breeding, fall migration, overwintering, and spring migration. To identify potential threats throughout the annual cycle, it is important to understand where, when, and how long a bird is present at each stage of the cycle [7, 8].

Conservation and management efforts for songbirds require an understanding of how populations are geographically linked through different phases of the annual cycle – a concept termed migratory connectivity [7, 9]. Individuals from populations of species with strong migratory connectivity show minimal geographic spread and little overlap with other populations through different phases of the annual cycle, whereas individuals from populations of species with weak migratory connectivity show greater geographic spread and may co-occur with individuals from different populations [7, 9]. Consequently, a threat on the overwintering grounds may have diffuse effects among multiple populations if weak migratory connectivity exists [7, 10]. However, it may be more challenging to implement effective conservation strategies across the annual cycle for populations with weak migratory connectivity.

In addition to studying population-level characteristics of a species, apparent annual survivorship is a valuable demographic rate because it can indicate the health of a population, life cycle stages at risk, and identify high-quality habitat [11, 12]. Survivorship is sensitive to short-term and local environmental changes and, therefore, can help elucidate the underlying mechanisms to changes in population size [11, 12]. However, robust annual survivorship estimates can be challenging to obtain as they require multiple years of data on marked individuals.

In this study, we examined the migration behaviour and estimated apparent annual survivorship of Gray Catbirds (*Dumetella carolinensis*) breeding west of and within the Rocky Mountain Range. Gray Catbirds are the only species within the Mimidae family whose migratory behaviour has been studied in detail but only in the eastern portion of its extensive North American range [13]. The known overwintering range of Gray Catbirds includes Florida, southeastern Texas, southeastern Louisiana, eastern Mexico, and Central America [14]. In the eastern and central portion of the breeding range, Ryder et al. [13] found catbirds breeding in the mid-Atlantic overwintered in Cuba and Florida, and those breeding in the Midwest overwintered in Central America. Likewise, stopover ecology is limited to eastern populations; one mark-recapture study in coastal Alabama during fall migration reported adult catbirds stayed on average for 4.1 days before departure [15].

The migration ecology of western Gray Catbirds, including how they travel through or around the Rocky Mountain Range, remains unexplored. Most migratory birds breeding west of the mountains migrate using the Pacific Flyway, whereas most birds east of the mountains follow the Central Flyway [16–19]. However, studies from central and coastal British Columbia tracked breeding Veeries (*Catharus fuscescens)* and Swainson’s Thrushes (*C. ustulatus)* across the Rocky Mountains to the Central Flyway towards overwintering sites in South America [20–22]. In 1964, it was postulated that western Gray Catbirds may migrate east before heading south, implying that these birds have to cross the Rocky Mountains [23]. Similarly, the analyses of stable hydrogen isotope ratios of catbird feathers linked overwintering birds in Mexico to inferred breeding sites located in the northwestern part of their breeding range [24].

The goals of our research on Gray Catbirds were to: (1) track migration routes; (2) identify stopover sites; (3) determine overwintering locations; (4) assess migratory connectivity; and (5) compare apparent survivorship for populations breeding in western Montana, USA, and southern British Columbia, Canada. Our research will verify whether western Gray Catbirds migrate using the Pacific Flyway or the Central Flyway. In addition, we will advance our understanding of the full annual cycle of western catbird populations and their migration ecology which may help ensure this species remains abundant on the landscape.

## Methods

### Study areas

We examined breeding Gray Catbirds in two locations: (1) the South Okanagan Valley, British Columbia, Canada (49.200° N, 119.552° W), and (2) the Bitterroot River Valley, Montana, USA (46.668° N, 114.023° W). These two study areas were 495 km apart, with the British Columbia population occurring west of the Rocky Mountains and the Montana population occurring within the Rocky Mountains. Catbirds were locally abundant in both study sites.

### Tracking devices

Gray Catbirds were captured in mist-nets passively or with the aid of call-playback in the breeding seasons from 2013 to 2018. Each bird was given a standard USGS band and a unique combination of colour-bands to aid in future identification. Birds were aged and sexed according to Pyle (1997) [25]. Adult male and female catbirds were outfitted with an M-Series light-level geolocator, Lotek, Newmarket, Canada (mass between 0.3–1.0 g, hereafter “geolocator”) or PinPoint GPS tag, Lotek, Newmarket, Canada, (mass 1.0 g, hereafter “GPS tag” [26, 27]). Geolocators were used only to infer general movement patterns whereas GPS tags were used for movement patterns plus quantitative analyses.

Tracking devices were fitted onto birds by creating a leg-loop harness which allowed the device to sit on the lower back of the bird, an optimal position for sunlight and satellite communication [28]. The harness was made of Stretch Magic jewelry cord manufactured by Pepperell Braiding Company, MA. Stretch Magic is an elastic-like, transparent, monofilament made of polyurethane and polyester and was ideal for accommodating fluctuations in size throughout the year. The harnesses were closed either by using a crimp bead or melting with a soldering iron, allowing harnesses to be custom fit to birds of varying sizes. Throughout the project, we checked recaptured birds for any sign of wear, abrasion, or feather loss and found minimal undesirable effects, however, some birds had evidence of light chafing on their thighs. Harnesses were durable and we had no instances of birds losing their geolocators or GPS tags with this method. This technique was adopted from other researchers who used the method successfully for attaching geolocators to songbirds [29, 30].

The average mass of Gray Catbirds was 36.3 g, therefore, tracking devices were < 3% of body mass, which is the preferred maximum for tracking birds and within the < 5% recommendation by animal care committee standards [31, 32]. Due to the small size of the devices, the number of GPS fixes were limited, with up to 8 fixes advertised for PinPoint-8 tags, up 10 fixes advertised for PinPoint-10 tags, and up to 80 fixes advertised for Swift PinPoint-10 tags [33]. Devices were retrieved in subsequent breeding seasons by targeting individuals identified by their unique colour-band combination. Captured birds had the harness and device removed then released unharmed. We deployed 39 geolocators and 21 GPS tags in British Columbia, and 32 geolocators and 52 GPS devices in Montana.

The average accuracy (± standard deviation) of the M-Series geolocator is 185 (±115) km, but many factors may affect the accuracy of geolocators, including shading, clouds, sensor degradation, and artificial lights [34]. The shrubby riparian habitat inhabited by Gray Catbirds may be an additional source of shade and further limit geolocator accuracy. The accuracy of GPS tags varies depending on the number of satellites available during a scheduled fix, however, in all cases should be better than 300 m [33]. We tested the GPS tags before deployment in British Columbia at a stationary location and found that 90% of the points were within 100 m of the test location, and many within a few meters. GPS tags were pre-programmed to obtain GPS coordinates at specific dates, which varied by location and year but generally were 1–5 days apart during migration and 10–30 days apart during winter.

### Repeat tracking

Three Montana birds were tracked in multiple years with separate devices. One female catbird was given a geolocator in 2012, another geolocator in 2013, and a GPS tag in 2014. One male catbird was given a geolocator in 2013 and a GPS tag in 2014. Another male catbird was given a GPS tag in 2016 and another GPS tag in 2017.

### Analyses of tracking devices

Geolocator files (.lig) were downloaded and light data were processed using R (*v 3.5.1* [35]). Methods for the analyses of the geolocators followed Lisovski et al. (2020) [36]. We used the GeoLight (*v. 2.0.1* [37]), TwGeos (*v.0.1.2* [38]), and adehabitatHR (*v. 0.4.16* [39]) packages. Twilights were determined using the *preprocessLight* function in the TwGeos package using a threshold value of 0.5 because there did not appear to be any nighttime light interference. Twilights were edited using the *twilightEdit* function from the TwGeos package (with ‘window’ set to 4, ‘outlier.mins’ set to 45, and ‘stationary.mins’ set to 25). Overwintering dates were defined as November 15 – March 1 to avoid the equinox by 3 weeks and to include times when birds would be on their wintering grounds [40]. The Hill-Ekstrom method was used to determine the sun elevation angle during winter months using the *findHEZenith* function from the TwGeos package [41, 42]. The Hill-Ekstrom method determines the correct sun elevation angle for stationary periods at unknown locations by determining a sun elevation angle that minimizes variance in latitude estimates [43]. Winter latitude and longitude estimates were calculated using the *coord* function of Geolight. To summarize the overwintering locations used by Gray Catbirds, 50% Kernel Density estimates were calculated from coordinates using the *kernelUD* function from the adehabitatHR package. A shapefile compatible with ArcMap 10.7.1 [44] was created by using the *getverticeshr* function.

The location coordinates from the GPS tags were vetted by removing points that had low accuracy, defined as those with dilution of precision values greater than 20 [33]. The remaining points were plotted using ArcMap 10.7.1. Points were connected to create migration tracks and to calculate distances, although we caution that these straight-line paths are our best approximation of migration route as the exact path is unknown. We defined stopover sites as locations with 2 or more consecutive fixes during migration outside of the mapped non-breeding range in September and early October. GPS fixes were programmed to be taken between 1 and 5 days apart, therefore, the minimum stationary period during migration that we considered a stopover was 24 h. Overwintering locations were defined as the first stationary period of consecutive fixes in the known overwintering range. However, for the older generation GPS tags where 8 or fewer fixes were obtained, we assume that the points occurring from mid-October onwards correspond to overwintering locations, as most GPS-tagged birds from both sites had reached their overwintering locations by early October based on the later model Swift GPS tags. To visualize potential travel routes around or within the Rocky Mountains, tracks and points were overlaid on a 30 arc-second digital elevation model of North America in ArcMap 10.7.1 [44]. To better contextualize habitat and elevation at stopover locations and overwintering locations, we overlaid satellite imagery using ArcMap 10.7.1 for each catbird during stopovers and in the non-breeding period.

### Migratory connectivity

Migratory connectivity was quantified with a Mantel’s correlation (r_M_) which involves comparing two matrices and their random permutations [45]. A significant positive correlation in the distances between breeding individuals and overwintering individuals suggests strong migratory connectivity [46]. Our matrices included (1) the geographic distances between all individuals on the breeding ground and (2) the geographic distances between all individuals on the overwintering grounds. Because some catbirds moved around during the overwintering period and we did not have data for the full overwintering period for all birds, we used the first overwintering location for the second matrix. Note that only geographic locations from GPS tags were included in the distance matrices and not locations obtained by geolocators due to the inherent low precision associated with geolocators. Matrices were created using the r.dist.earth function of the *fields* package (*v.9.8.6* [47]) in R. The Mantel correlation coefficient was calculated using the *mantel.rtest* function of the ade4 package (*v.1.7.13* [48]). A *P*-value associated with the correlation coefficient was calculated based on 9999 random permutations. In addition to Mantel’s correlation, we calculated the average pairwise distances between all individuals within the breeding and overwintering locations separately for Montana and British Columbia birds to quantify the geographic spread of each population.

### Apparent annual survivorship

We included both recaptured and resighted birds in our apparent annual survivorship analyses. In British Columbia, the data set spanned 2015 to 2019 and a total of 537 birds were colour-banded. Two study sites in British Columbia were used and effort included (1) intermittent target and passive mist-netting in conjunction with deploying and retrieving tracking devices; and (2) a standardized approach. Target and passive netting involved setting up 1–8 nets from before sunrise to early afternoon at the latest and occurred intermittently (~ 1–3 times a week) between May–August. Concurrently, 1–3 biologists searched for birds using binoculars and confirmed colour-band combinations using high-zoom digital cameras or binoculars. Effort was increased during the last year of the study in 2019, where at least 5 days a week, catbirds were resighted or recaptured from the end of May to early July. The standardized approach occurred once a week between May 1–September 15 using 10 mist-nets in a fixed location for 6 h and occurred in the first site only.

In Montana, the dataset spanned 2006 to 2018 and a total of 741 catbirds were colour-banded. Two study sites in Montana were used and effort included (1) intermittent target and passive mist-netting in conjunction with deploying and retrieving tracking devices; (2) a standardized approach, (3) intermittent banding for educational purposes (5 days total). Target and passive netting included 1–3 technicians working most weekdays between May 25–July 15 from sunrise to approximately 11:30 AM to resight and target net colour-banded catbirds between 2012 and 2018 at the first site only. Standardized MAPS banding efforts occurred from 2011 to 2018 at the first site and 2006–2018 at the second site and involved 10 mist nets open 6 h a day for 7 sample periods during the breeding season [49]. Educational banding occurred only at the second site.

Apparent annual survivorship was calculated using RMark (*v. 2.2.7* [50]) in R. Cormack-Jolly-Seber models [51, 52] were used, which included the apparent annual survivorship (ϕ) and detection probability (p) as model parameters. Assumptions of Cormack-Jolly-Seber models are that (1) every bird has the same probability of being recaptured/resighted at the next sampling period; (2) every bird has the same probability of surviving to the next sample period; (3) Colour-bands are not lost or missed, and; (4) sampling time is short (or instantaneous) relative to the interval in between sampling times. To assess the goodness-of-fit of our data to the assumptions of Cormack-Jolly-Seber models, we used the program RELEASE via RMark using the function ‘release.gof’. Goodness-of-fit was assessed by examining the fit of the global model. Because we were comparing the fit of multiple models with varying parameters, we used the model with the highest number of parameters as the global model, which contained sex as a covariate. There was no evidence of a lack of fit of the global model to Cormack-Jolly-Seber model assumptions (*χ*^2^ = 27.35, df = 25, *P*-value = 0.34).

Birds of all ages (except nestlings) were used in the analyses. Sex was included as a covariate for both survivorship and detection. To account for potential local differences between sites within each study area, site was included as a covariate. Because the first encounter is likely to include young or transient birds who are more likely to permanently leave the study area through death or immigration and thereby have a lower apparent annual survivorship in this first year after being colour-banded, we included a time-since-marking covariate where the first encounter is separated from subsequent encounters [53, 54]. Multiple models that contained different combinations of covariates were compared using an Akaike Information Criteria (AIC) approach adjusted for small sample sizes (Table 1, Burnham and Anderson 2002). We considered models with ΔAIC_c_ values of less than 2 to have substantial support [55]. We also calculated AIC_c_ weight and considered any model with a weight of greater than 0.90 as a clear top model [55]. For all analyses, mean and standard error are reported unless otherwise noted.

**Table 1:** Movement summary of Gray Catbirds (*Dumetella carolinensis*) fitted with GPS tags

						Fall					Spring				
Tag ID	Bird ID	Sex	Year	Fix	Loc.	Depart	Arrive	Length (Days)	Distance (km)	Speed (km/day)	Depart	Arrive	Length (Days)	Distance (km)	Speed (km/day)
**British Columbia**															
42000	YRGX	M	2017	23	NLE	Sep 6	Oct 12	36	3905	108	–	–	–	–	–
42005	YROX	M	2017	53	TX	Sep 10	Oct 12	32	3236	101	–	–	–	–	–
48964	BBRX	M	2018	46	SLP	Sep 10	Oct 16	36	4686	130	–	–	–	–	–
48961	BBXB	M	2018	48	TAM	Sep 12	Oct 14	32	4098	128	–	–	–	–	–
48965	BBYX	M	2018	51	TAM, SLP, HID	Sep 16	Oct 22	36	4171	116	–	–	–	–	–
48963	BBXD	F	2018	28	TAM*	Sep 6	Oct 16	40	4192	105	–	–	–	–	–
**Mean ± SE**								**35 ± 1.2**	**4048 ± 193.5**	**115 ± 4.9**					
**Montana**															
251	LLBkA	F	2014	3	TAM	–	Dec 7	–	3235	–	–	–	–	–	–
262	DBkAR	M	2014	4	TX	–	Dec 7	–	2746	–	–	–	–	–	–
245	RAWW	M	2014	2	TAM	–	Jan 7	–	3507	–	–	–	–	–	–
40958	LRYA	M	2016	8	TAM, HID	–	Oct 18	–	3072	–	–	–	–	–	–
48126	LRYA	M	2017	38	TAM, HID	Sep 14	Oct 19	35	3390	97	Apr 26	May 31	35	3501	100
48120	ODAS	M	2017	41	TAM, VER, HID	Sep 9	Oct 14	33	3358	102	May 3	May 31	28	3424	122
48121	OOAS	F	2017	22	VER	Sep 4	Oct 29	55	3911	71	–	–	–	–	–
48123	DOGA	F	2017	36	TAM, HID	Sep 9	Oct 14	35	3579	102	Apr 26	May 24	28	3764	134
48124	GOAS	F	2017	40	TAM, VER	Sep 19	Oct 19	30	3407	114	May 3	May 31	28	3677	131
48131	WWAS	M	2017	36	TAM, SLP, VER	Sep 9	Oct 14	35	4032	115	–	–	–	–	–
48133	RLGA	F	2017	37	TAM, PUE	Sep 9	Oct 14	35	3401	97	May 3	–	–	–	–
48134	ADBkW	M	2017	37	VER, HID	Sep 9	Oct 4	25	3760	150					
Mean ± SE**								**35 ± 3.2**	**3604 ± 76.2**	**106 ± 7.8**			**29 ± 2**	**3591 ± 78**	**122 ± 7.7**

*Last fix on October 16, assuming overwintering site but could potentially still be on migration

**Mean (± Standard Error) but not including length or speed for units with less than 10 fixes

Summary of GPS tags attached to Gray Catbirds in the South Okanagan Valley, British Columbia, Canada, and in the Bitterroot River Valley, Montana, USA with an emphasis on migration. Year is year GPS tag deployed. The fall depart date represents the last fix at the breeding site and the arrival date represents the first fix at the overwintering site. The spring depart date represents the last fix at the overwintering site and the arrival date represents the first fix back at the breeding site. Locations (Loc.) are abbreviated as Nuevo Leon (NLE), San Luis Potosi (SLP), Tamaulipas (TAM), Veracruz (VER), Hidalgo (HID), Puebla (PUE), and Texas (TX). Dashes indicate missing data: fall depart date is unclear from three Bitterroot River Valley catbirds due to first fixes occurring mid-migration; lack of spring migration data is due to premature battery exhaustion

## Results

In British Columbia, the return rate of birds with geolocators was 8 out of 39 (20.5%), and 7 geolocators were retrieved. The return rate of catbirds with GPS tags was 6 out of 20 (30.0%), excluding one deceased catbird found in the same year of deployment. All 6 GPS tags were retrieved. We experienced poor performance in the geolocator technology. One geolocator had light data for only 2 months, but all other geolocators contained data for the full year. Light levels recorded on the geolocators were low and inconsistent, making the determinations on sunrises and sunsets spurious (an example of low-quality geolocator data can be found in additional file 1). All location estimates were severely outside known overwintering distributions (examples of erroneous location estimates can be found in additional file 1). All GPS tags contained data but fewer than the advertised maximum number of 80 fixes.

In Montana, the return rate of birds with geolocators was 10 out of 32 (31.3%), and 6 geolocators were retrieved. The return rate of catbirds with GPS tags was 18 out of 52 (34.6%), and 14 GPS tags were retrieved. All geolocators contained usable data, but 2 GPS tags did not contain usable data.

### Migration routes

**Fig. 1:**
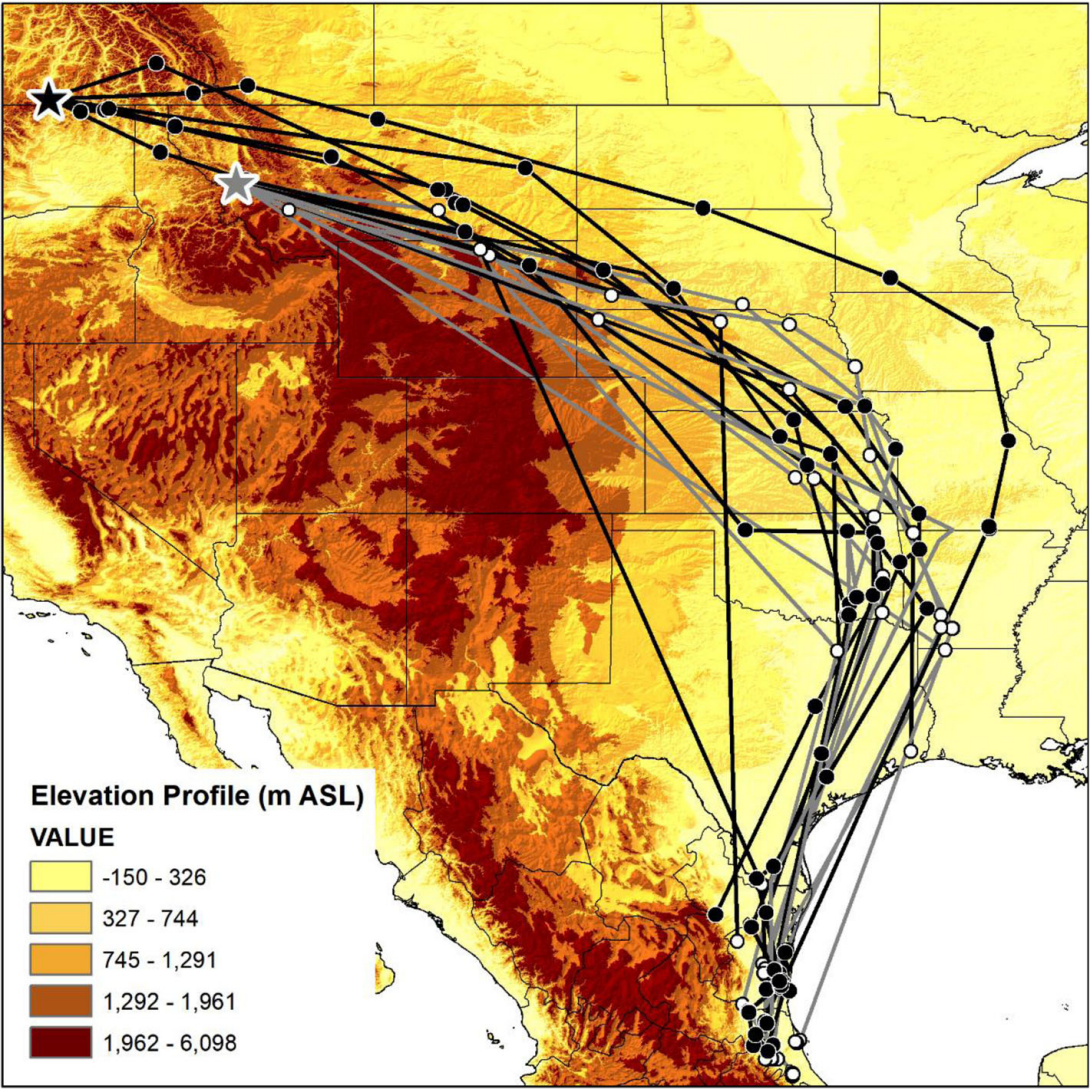
Gray Catbird (*Dumetella carolinensis*) fall migration route. Southward migration path from GPS tags retrieved from Gray Catbirds breeding in the South Okanagan Valley, British Columbia, Canada, and in the Bitterroot River Valley, Montana, USA. Gray tracks are from GPS tags retrieved from catbirds in the Bitterroot River Valley (*n* = 12) and black tracks are from GPS tags retrieved from catbirds in the South Okanagan Valley (n = 6). World Terrain Base Map provided by ESRI, USGS, and NOAA within ArcMap 10.7.1. Map created in ArcMap 10.7.1 (ESRI 2019) using the GCS WGS 1984 coordinate system. The elevation map was created in ArcMap 10.7.1 using the 30 arc-second digital elevation model of North America (ESRI 2019)

The migration route identified from GPS tags showed catbirds from Montana and British Columbia heading east across the Rocky Mountain Range, then south towards overwintering locations in Mexico and Texas (Fig. 1). While navigating the Rocky Mountain Range, Gray Catbirds from British Columbia appear to have traveled through lower elevation corridors; the elevation of points occurring within the Rocky Mountain Range were 1939, 849, 1020, 917, 497, 392, 662, 886, and 1034 m whereas the mountainous peaks within British Columbia, Washington, Idaho, and reach heights > 3000 m above sea level (Fig. 1). The fixes for Montana Gray Catbirds occurred too far east of the Rocky Mountain Range to assess their specific paths through the mountains.

**Fig. 2:**
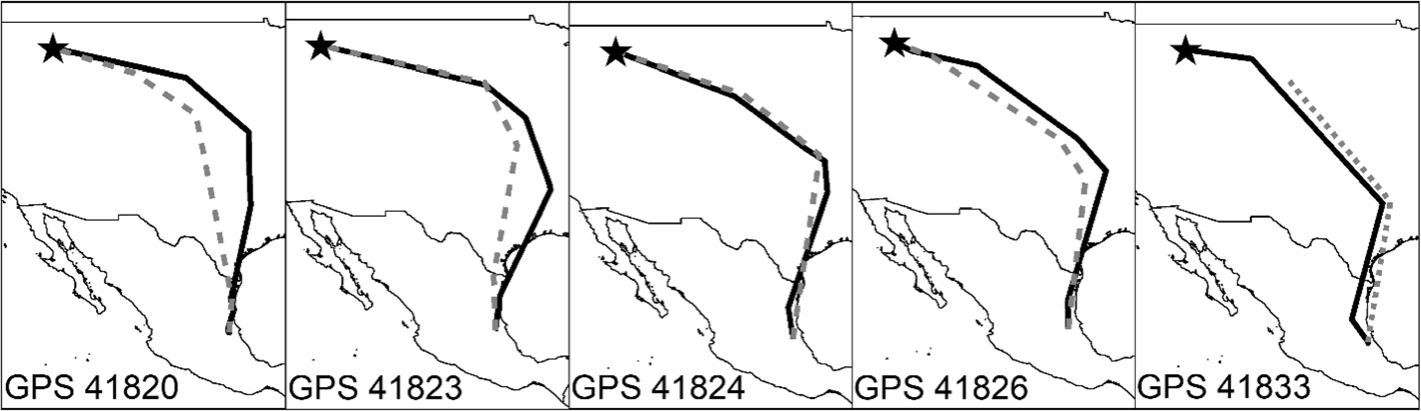
Comparing north vs. south migration routes for Gray Catbirds (*Dumetella carolinensis*). A comparison of southward (black) and northward migration (gray) GPS tracks from 5 different Gray Catbirds marked in their breeding site in the Bitterroot River Valley, Montana, USA. All birds were heading north by May 10, except for catbird with GPS 41820, who headed north by May 3. Map created in ArcMap 10.7.1 using the GCS WGS 1984 coordinate system (ESRI 2019)

In 5 cases from Montana, we have at least part of the spring migration route. These birds followed the same general routes in fall and spring (Fig. 2). Migration speed averaged 113 km/day and we saw no difference between spring and fall (t = − 1.3, df = 10, *P*-value = 0.24, 95% CI: − 43.7, 12.2; Table 1). No spring migration data was available from British Columbia catbirds due to GPS tag battery exhaustion.

### Stopover sites

**Fig. 3:**
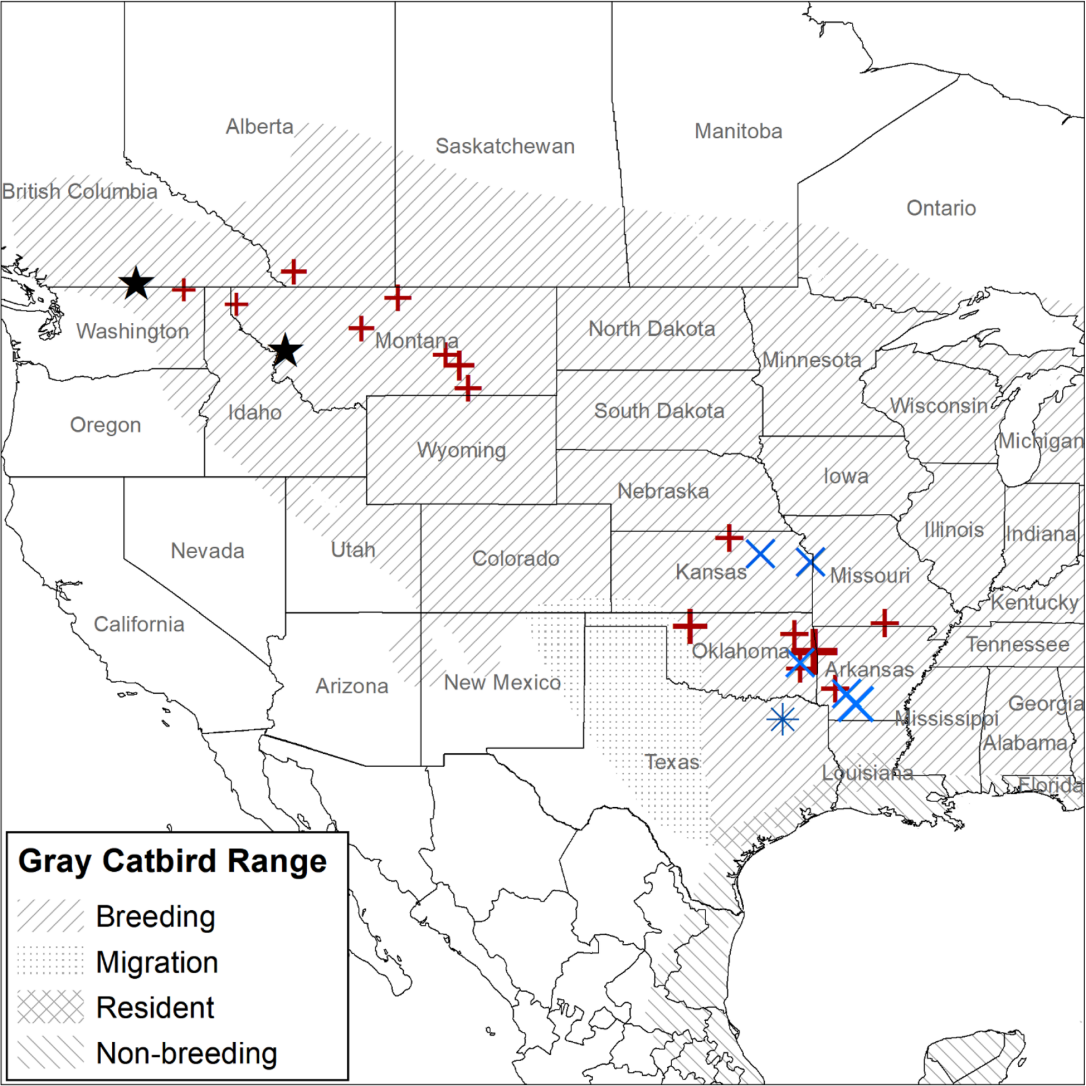
Migration stopover sites identified for Gray Catbirds (*Dumetella carolinensis*). Stopover sites identified using GPS tags. Crosses represent birds tagged in the South Okanagan Valley, British Columbia, Canada, and exes represent birds tagged in the Bitterroot River Valley, Montana, USA. Stars represent study site locations. All sites are from fall migration, except for the asterisk in Texas, which was a spring stopover site for a Gray Catbird that bred in the Bitterroot River Valley. Points are scaled relative to the amount of time spent at a site and range between 1 and 16 days. Gray Catbird range map provided by IUCN (2016). Map created in ArcMap 10.7.1 using the GCS WGS 1984 coordinate system (ESRI 2019)

We identified 20 fall stopover sites and one spring stopover site from our two catbird populations combined (Fig. 3). None of the tagged Gray Catbirds stopped at the same stopover site, and the closest sites were 24.7 km apart. Stopover sites for British Columbia Gray Catbirds were in Montana, Kansas, Oklahoma, Missouri, and Arkansas (Fig. 3). Stopover sites for Montana Gray Catbirds were in Kansas, Oklahoma, and Arkansas. The amount of time spent at each stopover site ranged between 1 to 16 days, but our estimate of stopover duration is limited by the sampling interval of the GPS tag ranging from 1 to 5 days during migration. From satellite imagery, stopover habitat generally included patches of trees or shrubby habitat, often within 500 m to water (satellite imagery of stopover sites can be found in additional file 2). The exception to the pattern of riparian habitat use was one stopover in an urban environment. The elevation of stopover sites varied widely, from 74 to 1060 m above sea level.

### Overwinter sites

**Fig. 4:**
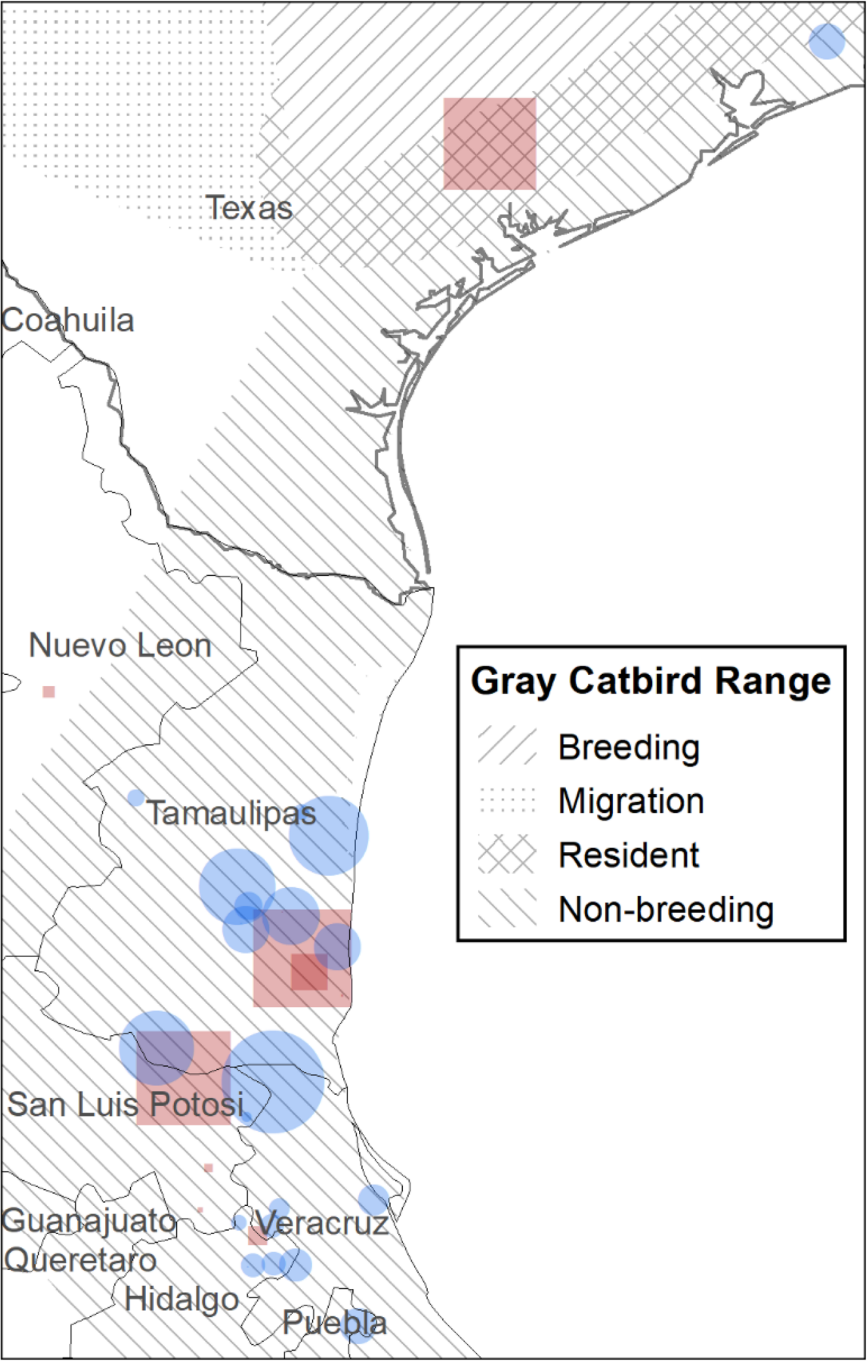
Gray Catbird (*Dumetella carolinensis*) overwintering sites. Overwintering points were determined from GPS tags retrieved from Gray Catbirds breeding in the South Okanagan Valley, British Columbia, Canada (red squares), and the Bitterroot River Valley, Montana, USA (blue circles). The size of the symbol represents how long the bird stayed at the site, ranging from 4 to 169 days. Most birds had more than one overwintering site. Gray Catbird range map provided by IUCN (2016). Map created in ArcMap 10.7.1 (ESRI 2019) using the GCS WGS 1984 coordinate system

**Fig. 5:**
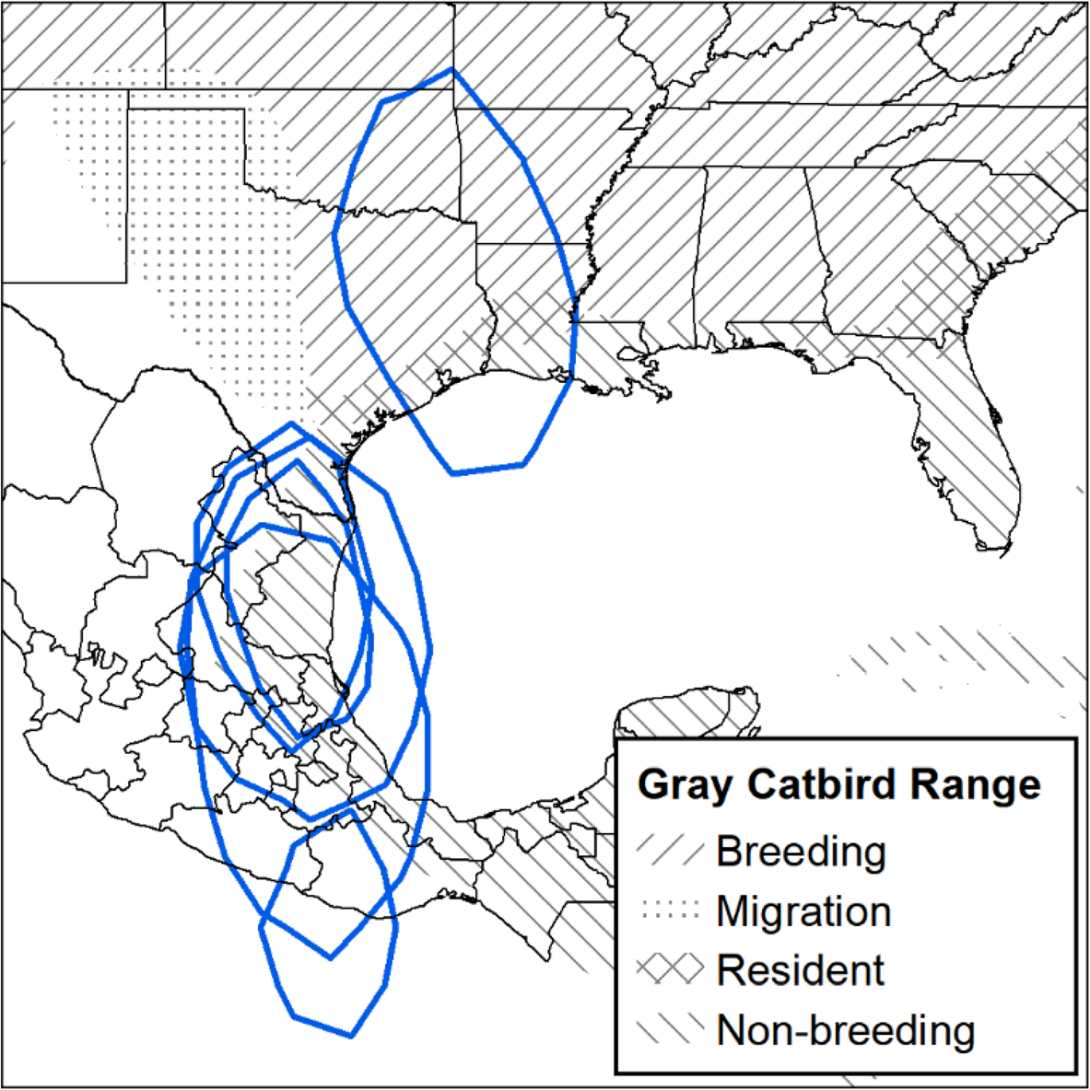
Overwintering locations of Gray Catbirds (*Dumetella carolinensis*) breeding in the Bitterroot River Valley, Montana, USA. Each polygon represents the 50% Kernel Density Estimates from locations obtained from archival light-level geolocators between November 15 – March 1 for 1 year from 2014 to 2018. Gray Catbird range map provided by IUCN (2016). Map created in ArcMap 10.7.1 (ESRI 2019) using the GCS WGS 1984 coordinate system

Gray Catbirds that bred in British Columbia and Montana had overlapping overwintering areas in Texas and Mexico. Gray Catbirds breeding in British Columbia overwintered in Texas, Tamaulipas, San Luis Potosi, Hidalgo, and Nuevo Leon, Mexico (Fig. 4 and Table 2). Gray Catbirds breeding in Montana overwintered in Texas, San Luis Potosi, Tamaulipas, Veracruz, Puebla, and Hidalgo. Gray Catbirds breeding in British Columbia traveled an average of 4048 ± 193 km to overwintering locations, and Gray Catbirds breeding in Montana traveled 3604 ± 76 km to overwintering locations. Overwintering location estimates from birds with geolocators were less precise than GPS tags but also encompassed San Luis Potosi, Tamaulipas, and Veracruz (Fig. 5). One exception is a Gray Catbird with a geolocator whose overwintering estimate was centered over Oaxaca, Mexico, and the Pacific Ocean - the longitude of this estimate is in accordance with all other Gray Catbird overwintering estimates but the latitude appears suspect (Fig. 5).

**Table 2:** Summary of Gray Catbird (*Dumetella carolinensis*) overwintering locations identified using GPS tags

	Winter Location 1			Winter Location 2				Winter Location 3				Winter Location 4			
Tag ID	Dates	State	Days	Dates	State	Days	KM	Dates	State	Days	KM	Dates	State	Days	KM
**British Columbia**															
42000	Oct 12-Nov 1	NLE	20												
42005	Oct 12-Mar 9	TX	158												
48964	Oct 16-Mar 27	SLP	162	Mar 31-Apr 10	SLP	10	109								
48961	Oct 14-Apr 2	TAM	169												
48965	Oct 22-Oct 26	TAM	4	Dec 1-Feb 1	TAM	62	32	Mar 1-Mar 17	SLP	16	178	Mar 19-Apr 20	HID	32	67
48963	Oct 16	TAM													
**Bitterroot River Valley**															
251	Dec 7-Jan 7	TAM	30												
262	Dec 7-Feb 7	TX	62												
245	Jan 7	TAM													
40958	Oct 18-Jan 5	TAM	79	Feb 4	TAM		19	Mar 1	HID		278				
48126+	Nov 1-Nov 21	TAM	20	Dec 1-Feb 19	TAM	80	5	Mar 15-Apr 26	HID	42	275				
48120	Oct 14-Nov 11	TAM	28	Dec 1-Feb 19	TAM	81	114	Mar 8-Apr 19	VER	41	234	Apr 26-May 3	VER	7	8
48121	Oct 29-Dec 21	VER	53	Dec 31	VER		6								
48123	Oct 14-Mar 1	TAM	138	Mar 15-Apr 26	HID	42	353								
48124	Oct 19-Mar 1	TAM	133	Mar 8-May 3	VER	56	313								
48131	Oct 14-Jan 20	TAM	98	Feb 19-Mar 8	SLP	17	168	Mar 22-Apr 26	VER	35	79				
48133	Oct 14-Feb 19	TAM	128	Mar 29-May 3	PUE	63	275								
48134	Oct 14-Mar 29	VER	176	Apr 5-May 3	HID	28	119								

+This bird moved around in Tamaulipas between Oct 19 and Nov 1

Details of overwintering sites obtained from GPS tags placed on Gray Catbirds in the South Okanagan Valley, British Columbia, Canada, and the Bitterroot River Valley, Montana, USA. Abbreviations include minimum length of stay (Days), the distance between current and previous overwintering site (KM). States are abbreviated as Nuevo Leon (NLE), San Luis Potosi (SLP), Tamaulipas (TAM), Veracruz (VER), Hidalgo (HID), Puebla (PUE), and Texas (TX)

From GPS tags, we found that several Gray Catbirds from Montana and British Columbia populations used more than one overwintering location, with up to four distinct locations documented (Table 2). Each subsequent overwintering location was farther south than the previous one, except one Gray Catbird who moved slightly farther north for his second overwintering location, then south for subsequent locations. Distance between overwintering locations ranged from 5 to 353 km. The length of stay at any one overwintering location ranged from 17 to 169 days. During winter, Gray Catbirds occupied natural areas with dense vegetative cover, often in association with edges or riparian habitats, at elevations of 9–678 m above sea level (satellite imagery of overwintering locations can be found in additional file 2).

### Multiple years of tracking

**Fig. 6:**
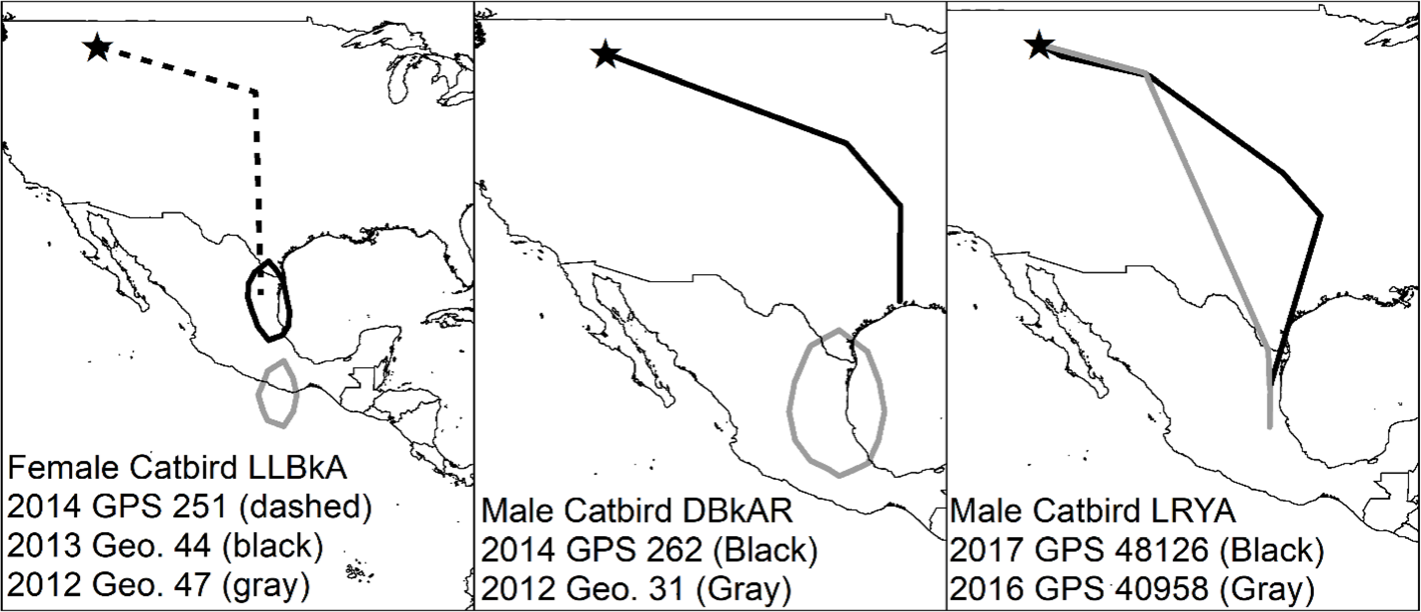
Repeat tracking of 3 separate Gray Catbirds (*Dumetella carolinensis*) in different years. Tracking devices were deployed in the Bitterroot River Valley, Montana, USA. The year that the track was recorded is shown. Map created in ArcMap 10.7.1 (ESRI 2019) using the GCS WGS 1984 coordinate system. Geolocator abbreviated as Geo

Some birds tracked over multiple years in Montana showed fidelity to overwintering locations (Fig. 6). One female Gray Catbird had two similar overwintering locations based on one GPS tag and one geolocator. This individual also had the potentially unreliable geolocator where latitude estimates appeared unlikely. One male Gray Catbird had likely different overwintering estimates, with one geolocator showing Mexico and one GPS tag showing Texas. Another male Gray Catbird showed strong overwintering fidelity over two years based on data from two GPS tags.

### Migratory connectivity

We observed weak migratory connectivity for British Columbia and Montana catbird populations; there was no significant correlation between the distances of birds in the breeding grounds and the distances of birds in the overwintering grounds (r_M =_ 0.013, *P*-value = 0.41). Additionally, the average pairwise distances in the overwintering grounds (British Columbia: 347.2 ± 68.3 km, *n* = 15, Montana: 255.8 ± 34.7 km, *n* = 66) were greater than the average pairwise distances in the breeding grounds (British Columbia: 0.74 ± 0.11 km, n = 15, Montana: 0.02 ± 0.002 km, n = 66), highlighting the degree of spread between different phases of the annual cycle.

### Apparent annual survivorship

The top model for apparent annual survivorship of Montana Gray Catbirds included sex and site and had a weight of 93% and no other model had an ΔAIC_c_ value of < 2, suggesting that this model had the greatest support (Table 3). Therefore, parameter estimates for this model alone are reported. The apparent annual survivorship was greater for males (ϕ = 0.43 ± 0.04) than females (ϕ = 0.34 ± 0.05, Table 4).

**Table 3:** Gray Catbird (*Dumetella carolinesis*) apparent annual survivorship model comparison

Model	Parameters	AIC_**c**_	ΔAIC	Weight	Deviance
**Montana (*n*** **= 741)**					
ϕ (sex) p (site)	5	496.4	0.00	0.93	159.9
ϕ (sex) p (.)	4	503.0	6.56	0.04	130.5
ϕ (site) p (sex)	5	503.7	7.27	0.02	167.2
ϕ (sex) p (sex)	6	506.8	10.36	0.01	130.2
ϕ (.) p (sex)	4	511.3	14.91	0.00	138.9
ϕ (TSM) p (sex)	5	513.3	16.94	0.00	138.9
ϕ (TSM) p (.)	3	552.4	56.00	0.00	65.2
ϕ (site) p (.)	3	596.4	100.00	0.00	124.3
ϕ (.) p (site)	3	596.5	100.14	0.00	124.4
ϕ (site) p (site)	4	598.2	101.76	0.00	124.0
ϕ (TSM) p (site)	4	598.6	102.17	0.00	124.4
ϕ (.) p (.)	2	600.7	104.20	0.00	115.4
**British Columbia (*n*** **= 537)**					
ϕ (sex) p (sex)	6	709.2	0.00	0.98	119.2
ϕ (sex) p (.)	4	718.1	8.91	0.01	132.1
ϕ (sex) p (site)	5	719.4	10.16	0.01	164.3
ϕ (site) p (sex)	5	724.0	14.82	0.00	169.0
ϕ (.) p (sex)	4	724.2	15.00	0.00	138.2
ϕ (TSM) p (sex)	5	726.1	16.91	0.00	138.1
ϕ (TSM) p (.)	3	813.4	104.24	0.00	51.1
ϕ (TSM) p (site)	4	815.4	106.22	0.00	76.4
ϕ (.) p (.)	2	836.7	127.48	0.00	76.4
ϕ (site) p (.)	3	838.5	129.34	0.00	101.6
ϕ (.) p (site)	3	838.6	129.45	0.00	101.7
ϕ (site) p (site)	4	839.7	130.48	0.00	100.7

Models for the apparent annual survivorship of Gray Catbirds in the South Okanagan Valley, British Columbia, Canada, and the Bitterroot River Valley, Montana, USA. Cormack-Jolly-Seber models were used, which include annual survivorship (ϕ) and detection probability (p). TSM is a time-since-marking approach whereby the first encounter is separated from separate encounters to factor in young and transient individuals. Intercept only models are designated by (.)

**Table 4:** Gray Catbird (*Dumetella carolinensis*) apparent annual survivorship model parameter estimates

Model	Parameter	Group	Estimate	Standard Error	
**Montana**					
ϕ (sex) p (site)	ϕ	Female	0.34	0.05	
ϕ (sex) p (site)	ϕ	Male	0.43	0.04	
ϕ (sex) p (site)	ϕ	Unknown	0.06	0.01	
ϕ (sex) p (site)	p	MPG Ranch	0.77	0.07	
ϕ (sex) p (site)	p	Other	0.34	0.10	
**British Columbia**					
ϕ (sex) p (sex)	ϕ	Female	0.61	0.06	
ϕ (sex) p (sex)	ϕ	Male	0.64	0.05	
ϕ (sex) p (sex)	ϕ	Unknown	0.20	0.20	
ϕ (sex) p (sex)	p	Female	0.25	0.07	
ϕ (sex) p (sex)	p	Male	0.72	0.07	
ϕ (sex) p (sex)	p	Unknown	0.35	0.13	

Parameter estimates for the top apparent annual survivorship models for Gray Catbirds in British Columbia and Montana. Cormack-Jolly-Seber models were used, which include annual survivorship (ϕ) and detection probability (p)

The top model for apparent annual survivorship of British Columba Gray Catbirds included sex and had a weight of 98% and no other model had an ΔAIC_c_ value of < 2. Therefore, parameter estimates for this model alone are reported. The top model for apparent annual survivorship in British Columbia included sex as a covariate for both survivorship and detection probability (Table 1). Males had greater detection probability (*p* = 0.72 ± 0.07) than females (*p* = 0.25 ± 0.07) and birds of unknown sex (*p* = 0.35) but males and females had similar survivorship (ϕ = 0.64 ± 0.05 and 0.61 ± 0.06, respectively, Table 4).

## Discussion

This study represents the first published information on migration, overwintering locations, and survival rates of western populations of Gray Catbirds. As such, it contributes to our collective knowledge of the species and provides baseline information against which to monitor future population change.

Our findings support past suspicions that western catbirds follow the Central Flyway, despite breeding locations within the Pacific Flyway. The Rocky Mountains did not appear to be a barrier during fall and spring migration. GPS data from British Columbia catbirds further highlight that low-elevation corridors are likely used to cross the mountainous landscape.

The migration route for western Gray Catbirds may reflect the evolution of migration in these populations during historical range expansion, similar to Veeries in British Columbia [22]. As is postulated for Veeries, Gray Catbirds likely originated in eastern North America and then slowly expanded their range northward and then westward as the continental glaciers receded [22]. The absence of this species in most of the Pacific Northwest, such as coastal Washington, Oregon, and California, is consistent with this hypothesis. The relicts of historical range expansions are recapitulated in migration and can be observed in other species whose breeding and overwintering grounds are continents apart, such as Northern Wheatear (*Oenanthe oenanthe* [56]), Bar-tailed Godwit (*Limosa lapponica* [57]), and Blackpoll Warbler (*Setophaga striata* [58]). In British Columbia, ancestral routes similar to what we have found in the western Gray Catbird have also been observed in Swainson’s Thrushes [20, 59], but our study is the first to document this phenomenon in the Mimidae family.

Ancestral relicts of range expansion may also work in conjunction with ecological limitations to explain why the migratory routes we documented are indirect and farther east than we expected [60]. Gray Catbirds occupy riparian habitat and shrubby edges throughout their range [40, 61]. Riparian habitats identified at our stopover locations suggest these cover types may be of particular importance during migration by providing both food and water, as has been documented for other bird species [4, 62, 63]. Indeed, fall stopover sites appear concentrated after migrating along the edge of the semi-arid great plains and reaching the temperate forest ecoregion, replete with wetlands, forests, and a temperate climate [64]. Therefore, catbirds taking the easterly route may have experienced an evolutionary advantage by spending more time in higher quality stopover habitat even as their range expanded westward.

In addition to confirming that catbirds use riparian and edge habitats for stopovers, we also found stopover locations dispersed on the landscape and not used repeatedly or by the same birds, though we acknowledge we have a relatively small sample size and this topic merits further study. Dispersed and abundant stopover sites pose a challenge for conservation as the persistence of many small riparian areas along the migration route may be necessary to support current Gray Catbird populations. Alternatively, perhaps the loss of any one patch of habitat is less consequential as long as a certain threshold of suitable habitat is maintained at the landscape scale. We suggest additional study into important features of stopover locations and to what extent catbirds are plastic in their use of such sites. Conserving a network of riparian habitats across the landscape is a valuable conservation approach for not just Gray Catbirds, but other wildlife, as it allows for landscape-level connectivity and the retention of movement corridors.

Birds from both of our study populations overwintered along the Gulf Coast of Texas south into northeastern Mexico, with the highest concentration in the Tamaulipas region. These sites do not overlap with known overwintering locations of Midwestern and mid-Atlantic catbirds [13] and thus represent a major advance in our understanding of the precise geographic linkages between breeding and overwintering catbird populations. We also saw some evidence of overwintering site fidelity, though potentially erroneous geolocator data limits our ability to confirm this behaviour across many individuals. In any case, repeated tracking of individual songbirds over multiple years is rare and, even if limited by sample size and technology, represents a major accomplishment of our study.

Our study is the first to document Gray Catbird’s use of multiple overwintering locations; we found individuals used multiple overwintering locations and moved farther south as the winter progressed into spring. The use of more than one overwintering location has been observed in a few other neotropical migrants (e.g. Prothonotary Warblers (*Protonotaria citrea*), Veeries, and Swainson’s Thrushes [22, 65–67]). Future tracking studies on additional species would help us understand if this behaviour is rare or common amongst neotropical migrants. The mechanisms driving overwinter movement warrants further research; plausible explanations may be that birds follow a shift in seasonal food resources or avoid weather events. A better understanding of overwintering habitat use and how it may vary by age and sex is a priority for future Gray Catbird research [40]. The ability of individuals to exhibit some plasticity in overwintering locations, suggests a life-history strategy adapted to landscape change. From a conservation perspective, the potential plasticity of overwintering site use may be an adaptive strategy that will bode well for the future of catbird populations to persist as habitats shift due to anthropogenic or natural causes.

That Gray Catbirds use multiple overwintering locations and vary in their degree of overwintering location fidelity question important assumptions of overwintering monitoring programs, such as Monitoreo de Sobreviviencia Invernal (MoSI), which uses standardized mist-netting efforts during the overwintering period to assess overwintering survivorship [68]. The MoSI protocol involves 5 monthly overwinter mist-netting pulses from November to the end of March where each pulse occurs over 2–3 days [69]. The Gray Catbird was one target species for the MoSI program but their overwintering survivorship estimates will likely be underestimated given their overwinter movements [69]. A better understanding of songbird overwintering movement and site fidelity in other species is crucial to assessing the accuracy of vital rate metrics derived from MoSI data.

### Migratory connectivity

We found weak migratory connectivity for the two Gray Catbird populations we examined, with Montana and British Columbia Gray Catbirds overwintering in similar areas across northeastern Mexico. Birds that bred close to one another dispersed up to 1000 km apart during the overwintering period. Weak migratory connectivity may be advantageous for the persistence of western catbird populations because individuals are dispersed across a large geographic region during the overwintering period are unlikely to be affected by local threats.

One challenge in comparing migratory connectivity between studies is the scale in which it is examined. If we consider both breeding sites from British Columbia and Montana as one population, and the span of Texas to Veracruz as one overwintering population, it would be perceived as strong migratory connectivity. Ryder et al. [13] reported strong migratory connectivity for eastern and central populations of Gray Catbirds. Regardless, the results of our study and previous studies on migration patterns of Gray Catbirds result in a comprehensive picture of population connectivity across their range. Gray Catbirds breeding in the west overwinter in Texas and northeastern Mexico [this study and 94]. Catbirds breeding in the mid-west overwinter in Central America [13], Catbirds breeding near the Great Lakes overwinter in Guatemala [70] and Catbirds breeding in the Atlantic overwinter in Cuba and the Caribbean Islands [13].

### Apparent annual survivorship

Establishing demographic rates for species, such as apparent annual survivorship, are important for identifying populations at risk of decline and informing conservation efforts [11, 71]. The estimates for Gray Catbirds in British Columbia (0.61 ± 0.06 female, 0.64 ± 0.05 males) exceed reported adult average survivorship values in the Northern Rockies bird conservation region (which spans both study areas) of 0.54 ± 0.02 between 1992 and 2006 [72]. For comparison, apparent annual adult survivorship for other common songbirds in the Northern Rockies bird conservation region ranges between 0.50–0.59 [72]. Why the apparent annual survivorship estimates for Montana Gray Catbirds (0.34 ± 0.05 females, 0.41 ± 0.04 males) are lower is unclear. However, when averaged across both sites, survivorship values match those of the general Northern Rockies conservation region, so perhaps the range of values we are documenting at local sites are in line with regional trends. Because birds from both study areas traveled along similar routes and overwintered in similar locations, differences are likely attributed to breeding locations. Breeding site fidelity in catbirds is linked with reproductive success, where birds that successfully reproduce are more likely to return to the same territory, therefore probing deeper into nest success in Montana might provide more insight into apparent annual survivorship trends [73]. Additionally, the degree of urbanization between the two sites may be relevant, as eastern catbirds exhibit lower survival in rural than urban areas (suspected due to food availability), and our Montana site was more rural than our British Columbia site [74]. Both study areas had resighting efforts, which when combined with recapture methods, improves apparent annual survivorship estimates [75]. However, overall resighting effort in Montana was slightly lower than in British Columbia, and therefore, could have skewed the results. As technology advances, we are hopeful that one day we will be better able to understand whether birds that do not return to the site in subsequent years are dispersing to new areas, remain undetected, or are dying.

## Conclusions

Our study represents the first description of full annual cycle movements of western populations of Gray Catbirds, revealing that catbirds breeding in two disparate populations followed similar migratory routes eastward across the Rocky Mountain Range then southward towards overwintering locations in northeastern Mexico and Texas, following a putative ancestral route. During the non-breeding season, individuals from both breeding populations spread out across overlapping geographic areas, a case of weak migratory connectivity. Unexpectedly, Gray Catbirds used multiple overwintering locations which suggests some plasticity in movement that may help catbirds remain adaptable if landscapes change. Like many previous catbird studies, our research found that western catbird populations use riparian and shrubby plant communities for breeding, stopover sites, and overwintering sites.

From a conservation perspective, the detail we now have on the full annual cycle of Gray Catbirds may help in ensuring the species remains a common species on the landscape well into the future. Preserving key areas of suitable habitat in the central and southern portions of Tamaulipas would benefit both Montana and British Columbia Gray Catbirds because our study identified Tamaulipas as a key overwintering area. Additionally, with the predicted effects of climate change impacting the persistance and quality of riparian habitats, proactively protecting and monitoring riparian areas along their migration route and stopover areas may allow us to detect changes early [76, 77]. Moreover, should a population decline occur in the future, the baseline information on the survivorship and migratory connectivity of these populations we describe in detail will be valuable for conservation efforts [78].

## Supplementary Information

**Additional file 1.** Low-quality geolocator results. Additional file 1 shows an example of low-quality archival light-level geolocator data and the associated geographic assignment locations.

**Additional file 2 **Aerial imagery of stopover and overwintering sites. Shows satellite imagery of stopover and overwintering site locations as determined from GPS tags attached to Gray Catbirds (*Dumetella carolinensis*).

## Data Availability

The datasets supporting the conclusions of this article will be available in the Open Science Framework repository.

## Abbreviations

AIC: Akaike’s Information Criteria
GPS: Global Positioning System
MoSI: Monitoreo de Sobreviviencia Invernal
r_M_: Mantel’s correlation
USA: United States of America
