# Supplementary material for: Migration ecology of western gray catbirds
Source: Mov Ecol. 2021 Mar 17;9:10. doi: 10.1186/s40462-021-00249-7 (PMC7972347; doi:10.1186/s40462-021-00249-7)
Supplement: Supplementary file 1 — Additional file 1. Low-quality geolocator results. Additional file 1 shows an example of low-quality archival light-level geolocator data and the associated geographic assignment locations. [file 40462_2021_249_MOESM1_ESM.docx]

**Additional file 1 -** Low-quality geolocator results

The quality of the data varied drastically between geolocators deployed on Gray Catbirds (*Dumetella carolinensis*) in the South Okanagan Valley, British Columbia, Canada and the Bitterroot River Valley, Montana, USA (Figure 10). The overwintering location estimates obtained from geolocators on catbirds in the South Okanagan Valley were substantially farther north than their known overwintering range. Shading affects location estimates (Lisovski et al. 2012) and likely explains our results. While the longitude estimates are robust to shading, and are in line with the known overwintering range, the large error in latitudes (>600 km, Figure 11) provides little to no additional useful information especially when compared to the precise overwintering estimates we have retrieved from GPS tags. At best, the longitudinal estimates suggest that South Okanagan Valley catbirds generally overwinter in the western part of their overwintering range.


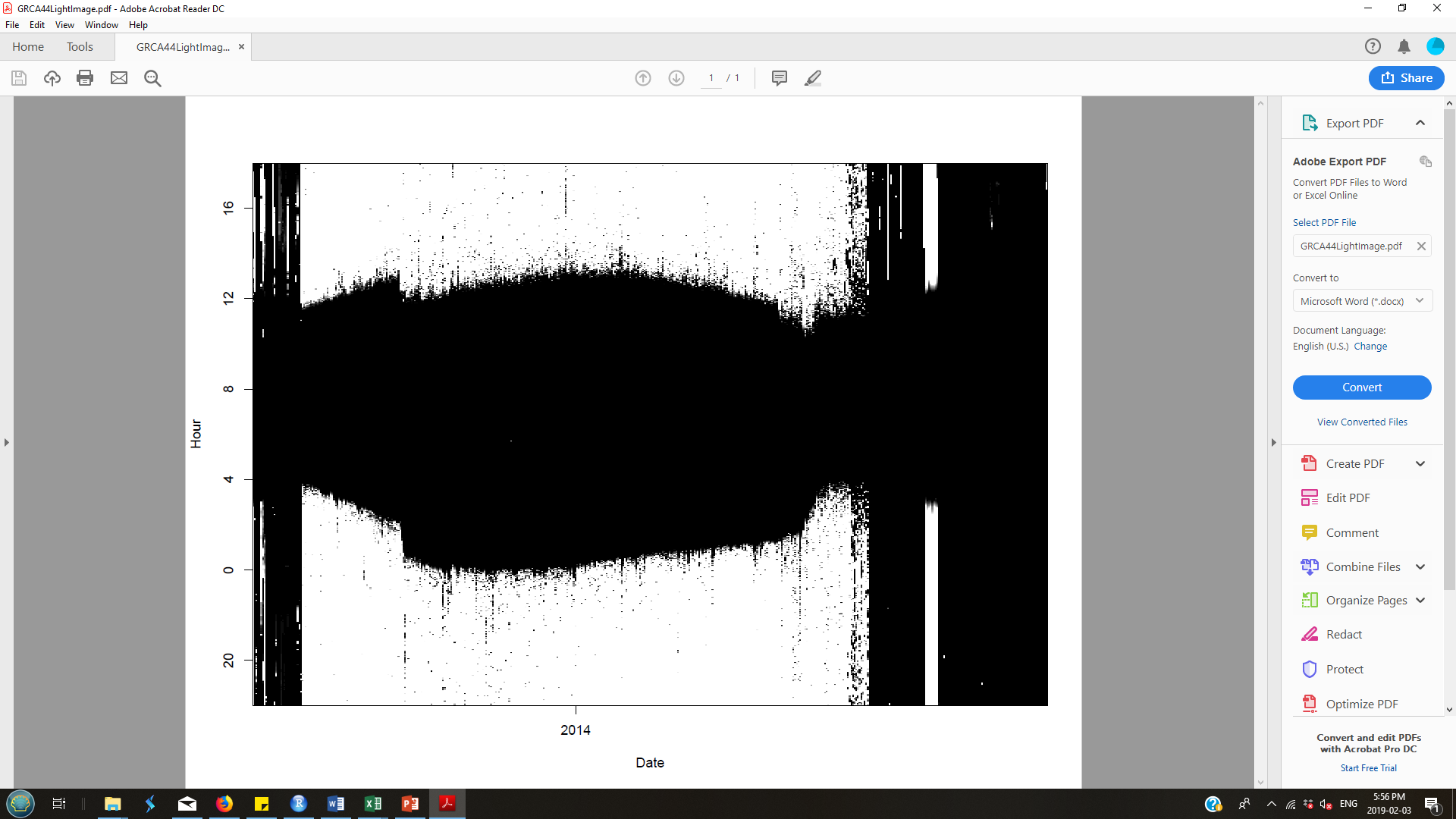

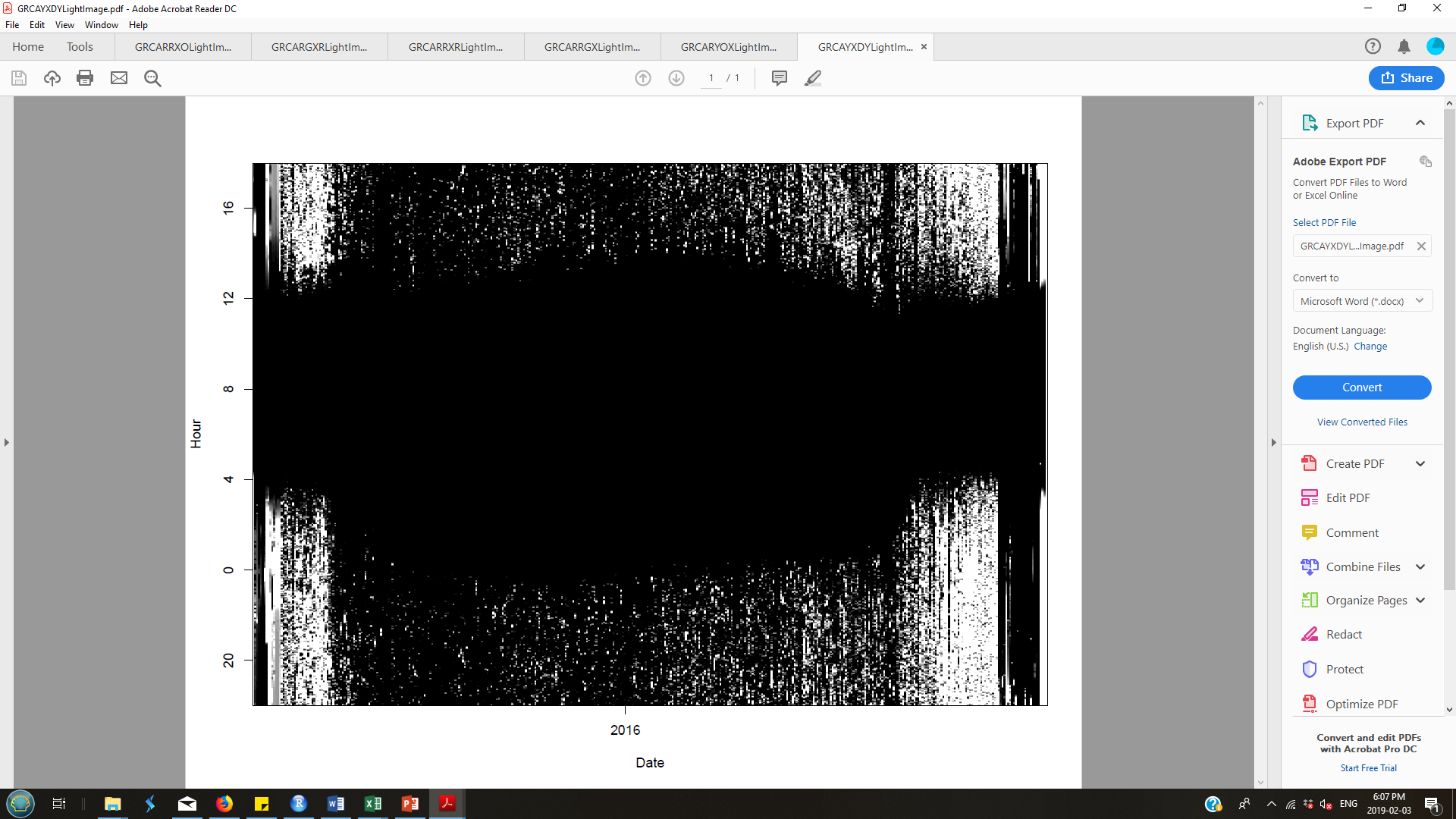


**Figure 10**. A comparison between high quality and low-quality light images from geolocators on Gray Catbirds (*Dumetella carolinensis*). The left image is from a geolocator on a catbird from the Bitterroot River Valley, Montana, USA, whereas the image on the right is from a geolocator on a Gray Catbird in the South Okanagan Valley, British Columbia, Canada.


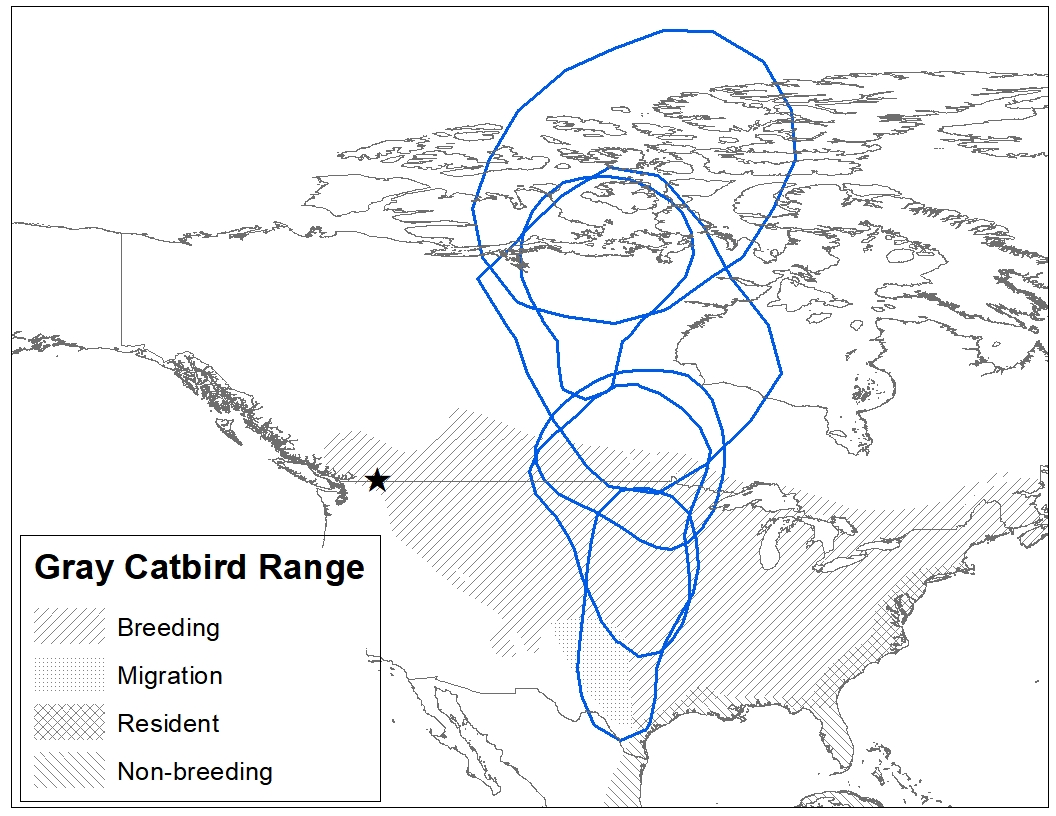


**Figure 11**. Geolocator overwintering location estimates from Gray Catbirds (*Dumetella carolinensis*) breeding in the South Okanagan Valley, British Columbia, Canada. Study area is indicated by the star. Each polygon represents 50% Kernel Density Estimates from predicted locations between November 15 – March 1. These estimates do not fall within the known wintering range of the species and do not corroborate the results from precise GPS tags. The Gray Catbird range map was from IUCN (2016). The Map was created in ArcMap 10.7.1 (ESRI 2019) using the GCS WGS 1984 coordinate system.
