# Supplementary material for: Migration ecology of western gray catbirds
Source: Mov Ecol. 2021 Mar 17;9:10. doi: 10.1186/s40462-021-00249-7 (PMC7972347; doi:10.1186/s40462-021-00249-7)
Supplement: Supplementary file 2 — Additional file 2 Aerial imagery of stopover and overwintering sites. Shows satellite imagery of stopover and overwintering site locations as determined from GPS tags attached to Gray Catbirds (Dumetella carolinensis). [file 40462_2021_249_MOESM2_ESM.docx]

**Additional File 2 –** Aerial imagery of stopover and overwintering sites

Aerial imagery of stopover and overwintering sites used by Gray Catbirds (*Dumetella carolinensis*) from birds that were breeding in the south Okanagan Valley, British Columbia or the Bitterroot River Valley of western Montana. Sites were determined based on GPS tags attached to Gray Catbirds. Note that for stopover sites, all points are during fall migration, except for one point.

**Stopover sites**

| 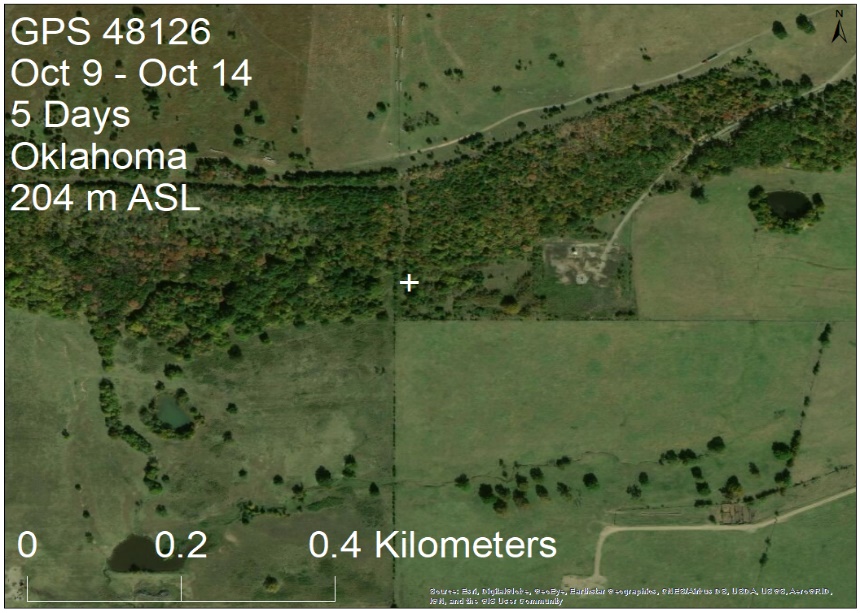 | 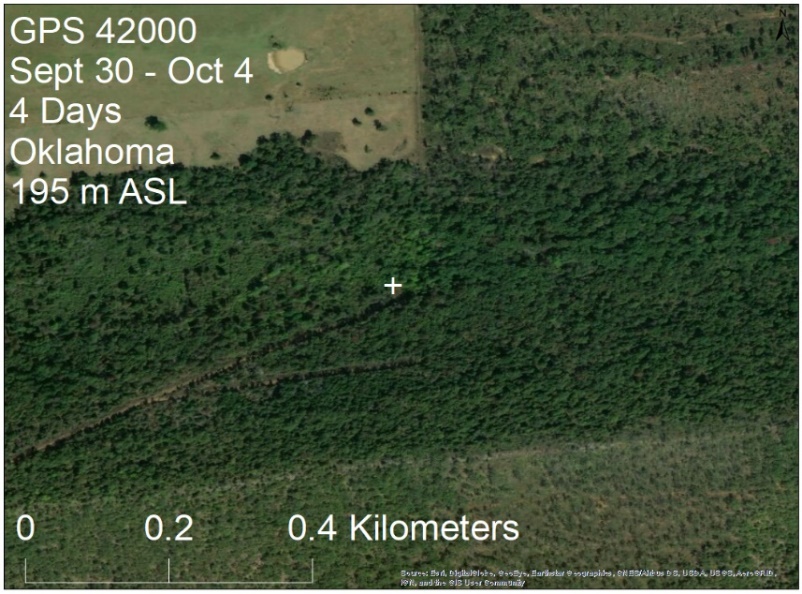 |
| --- | --- |
| 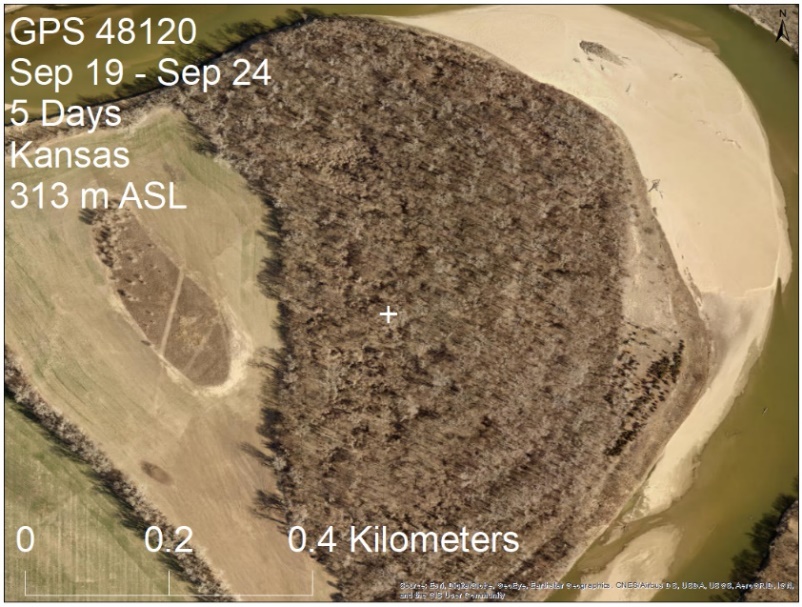 | 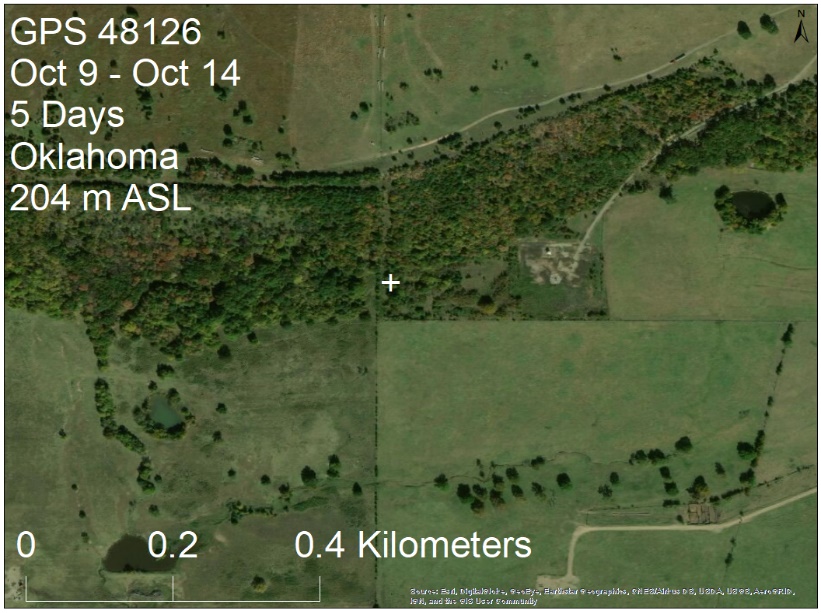 |
| 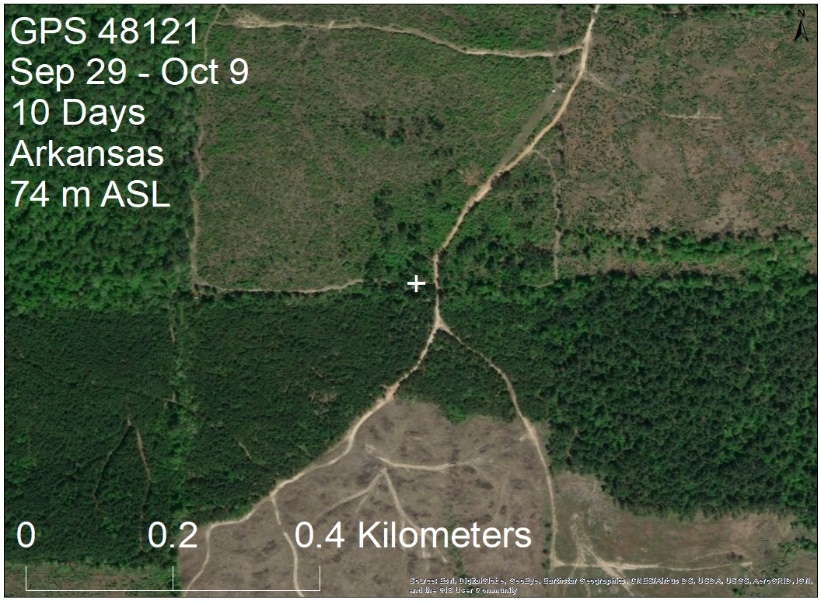 | 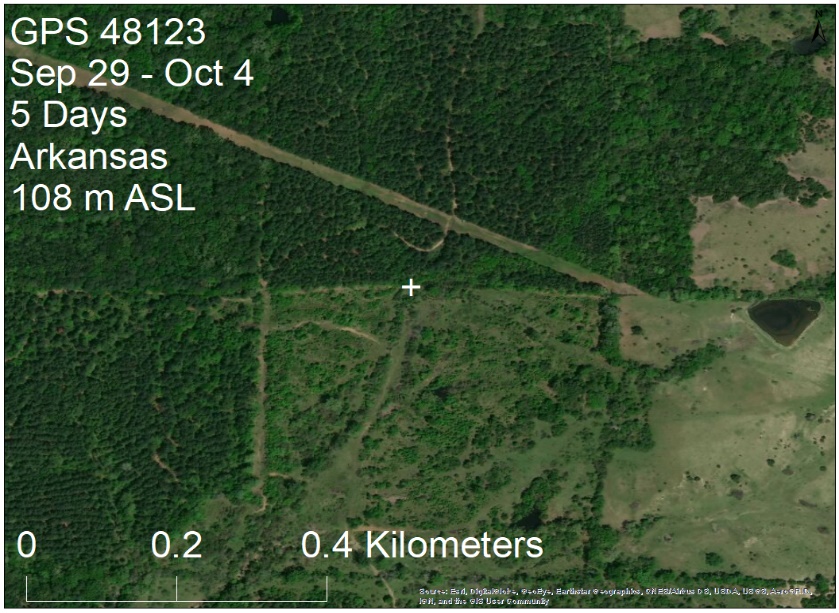 |
|  |  |
| 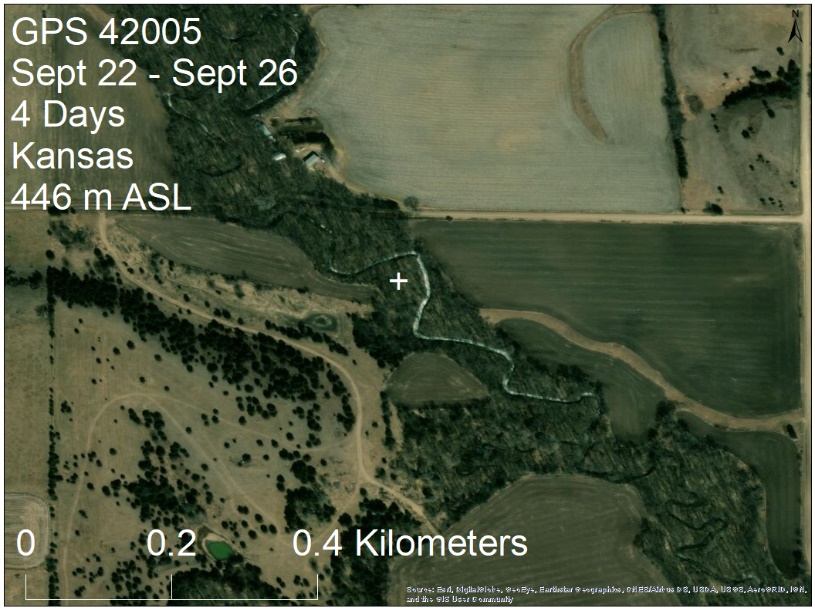 | 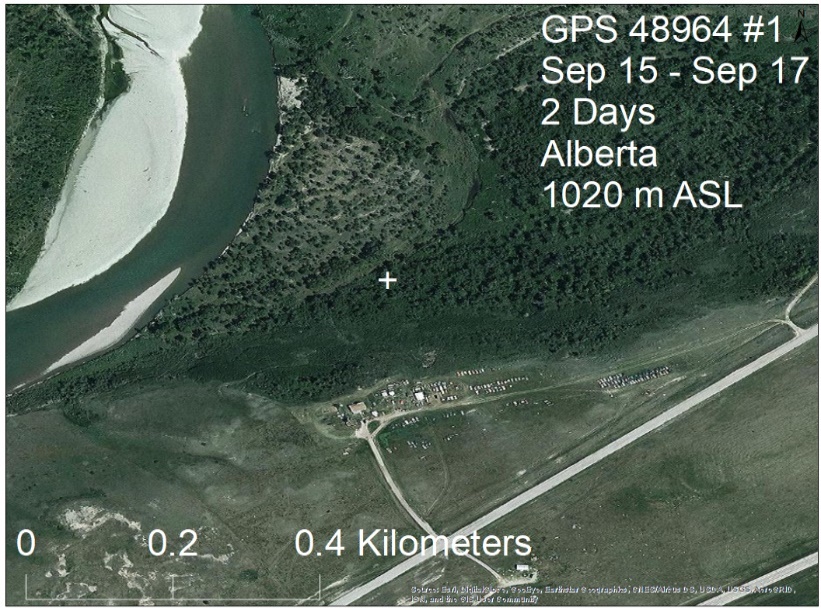 |
| 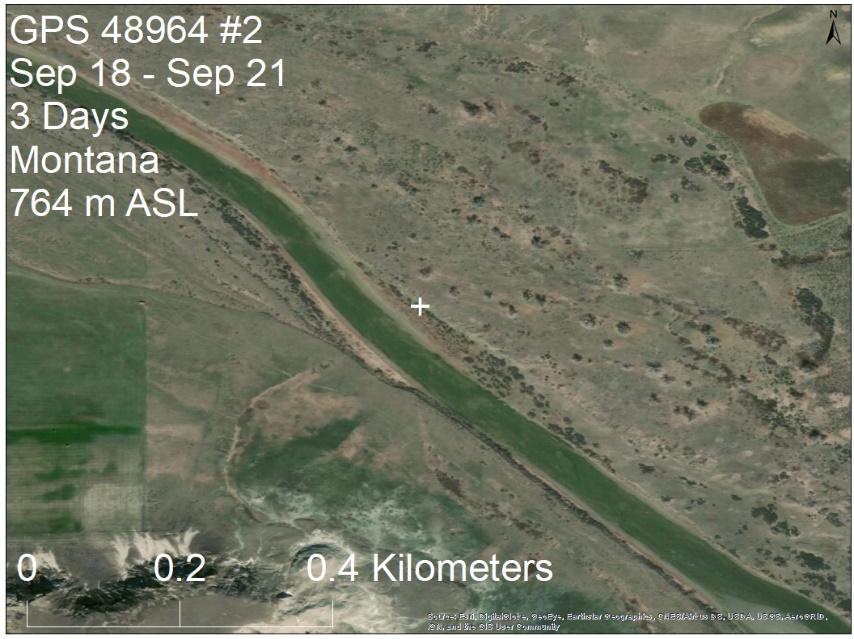 | 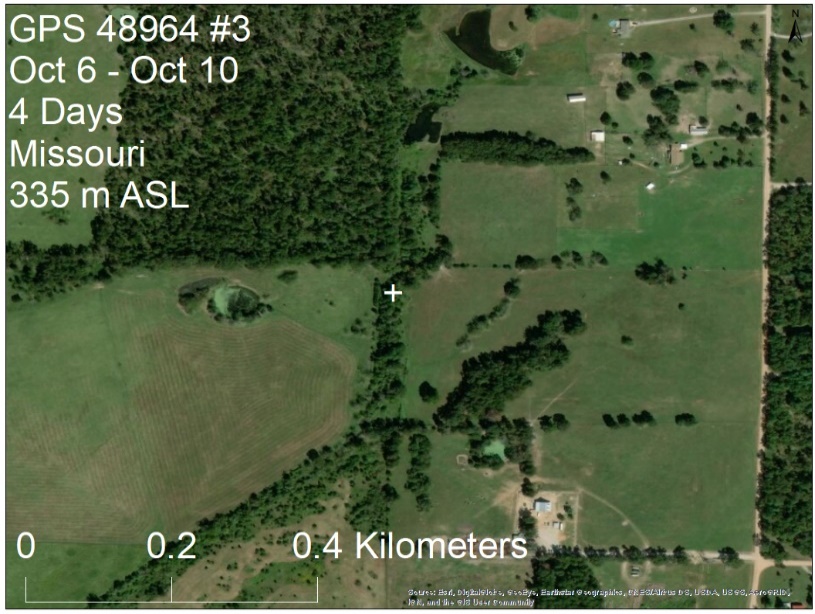 |
| 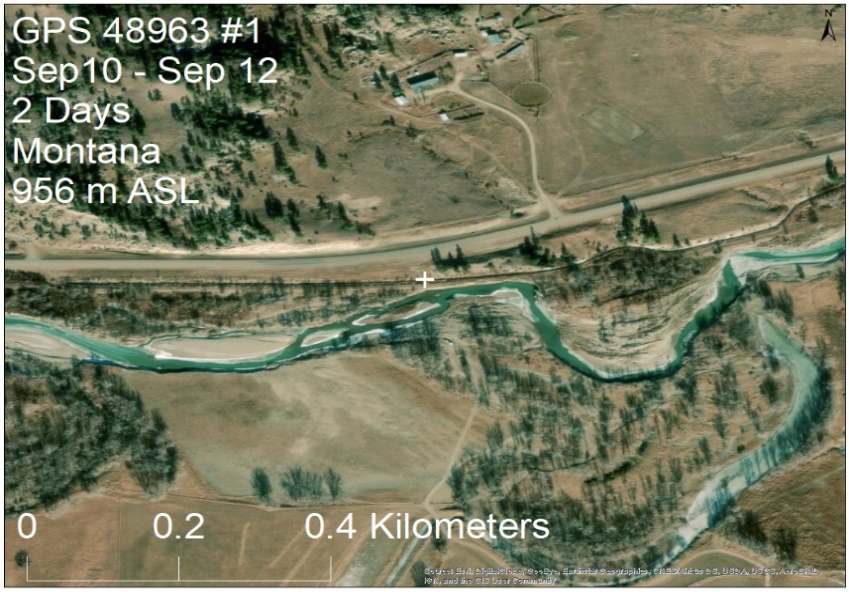 | 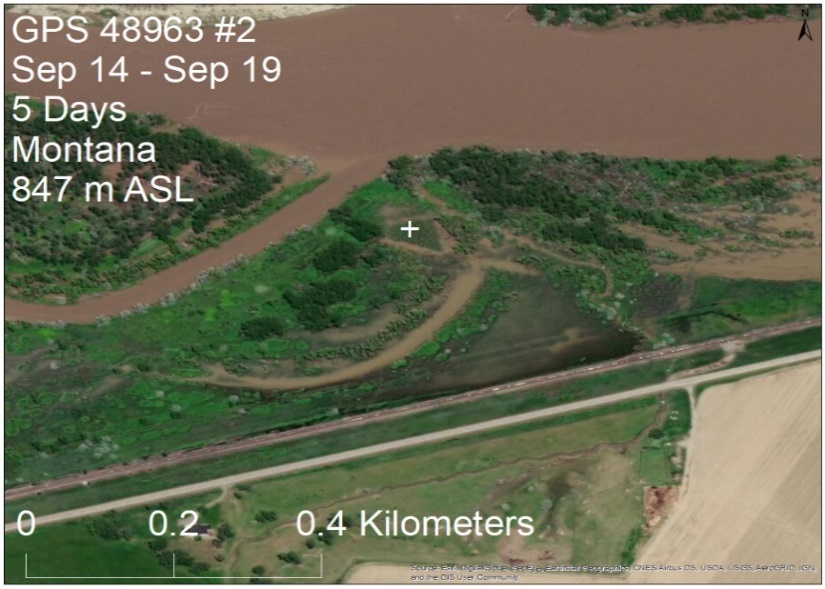 |
| 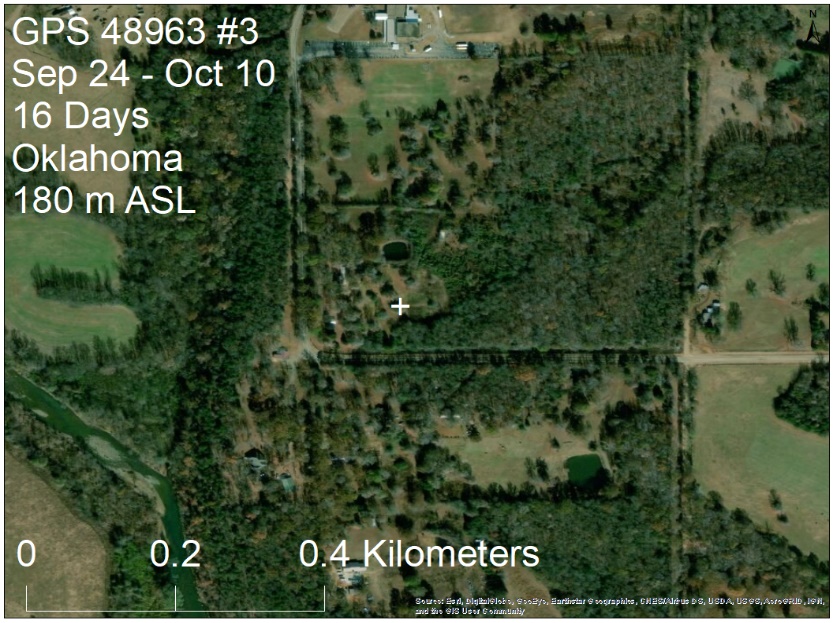 | 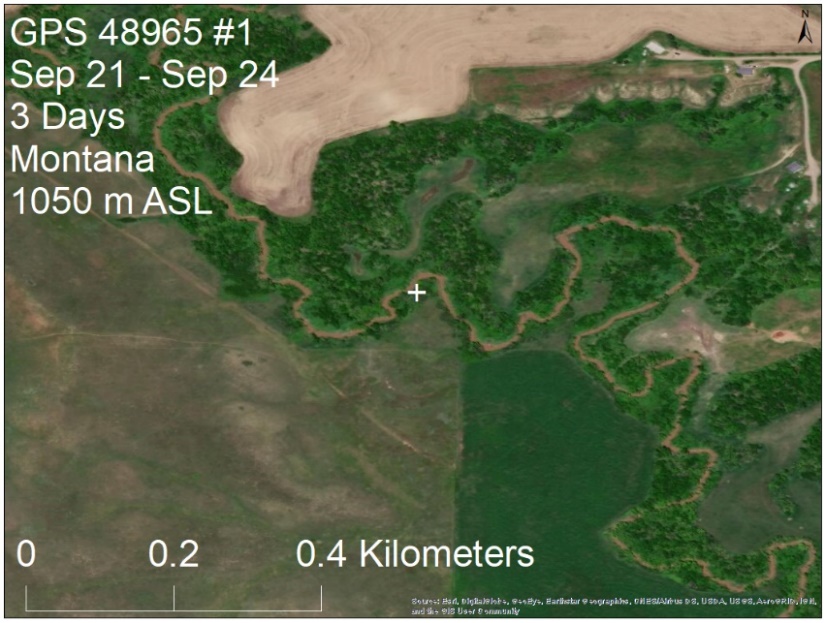 |
| 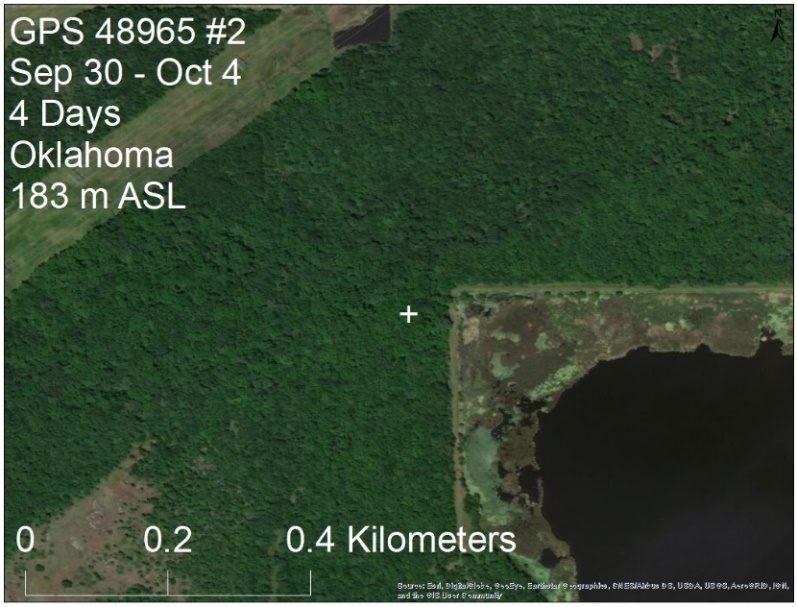 | 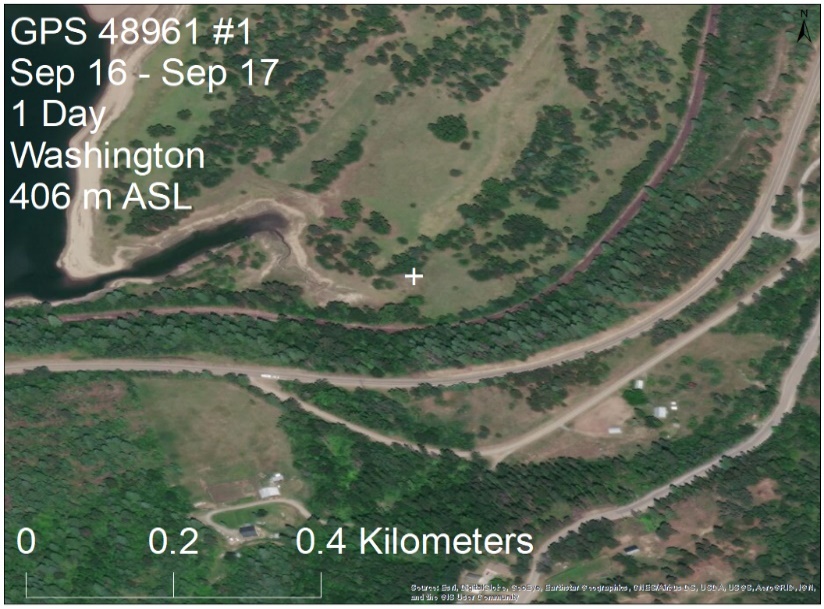 |
| 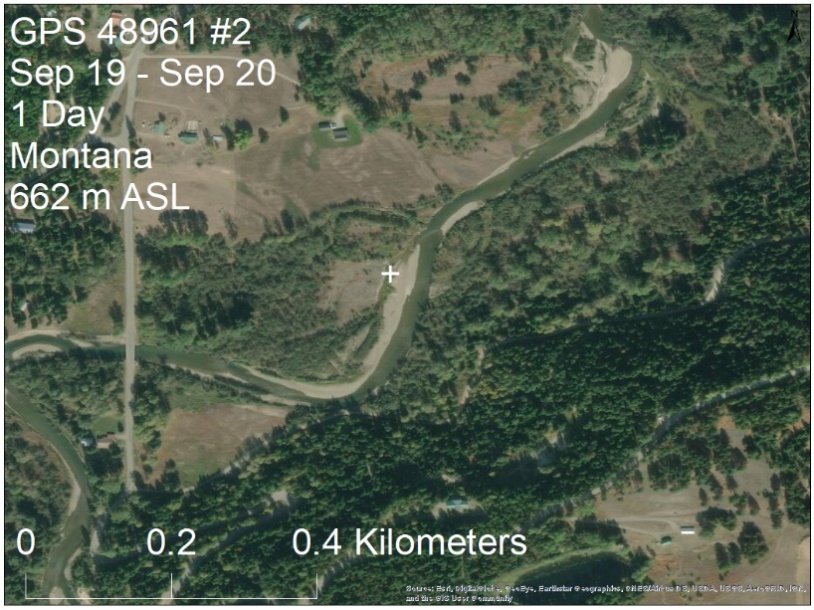 | 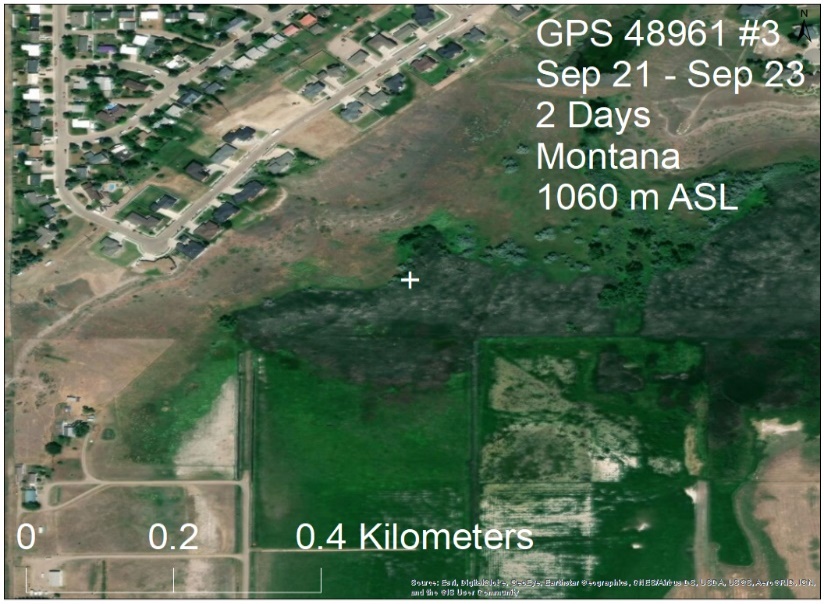 |

| 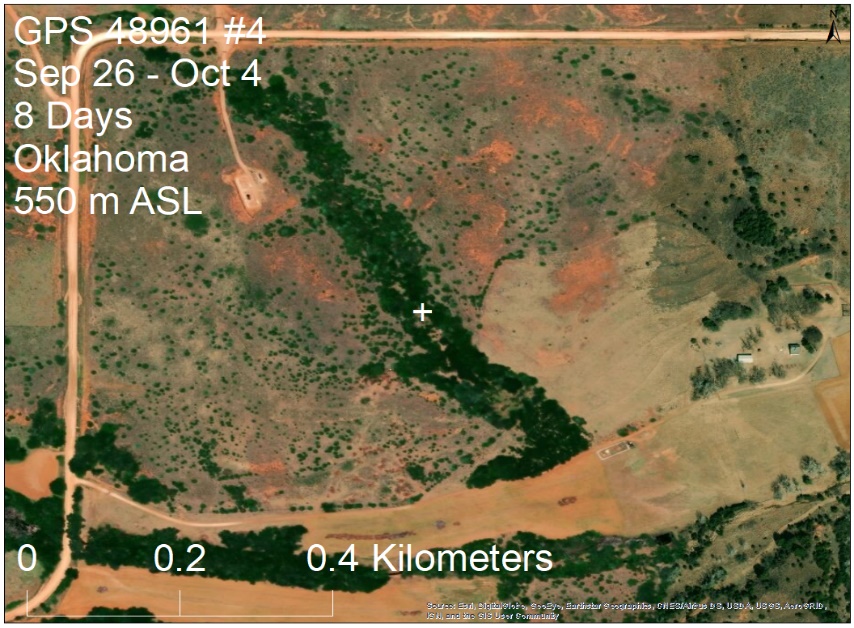 | 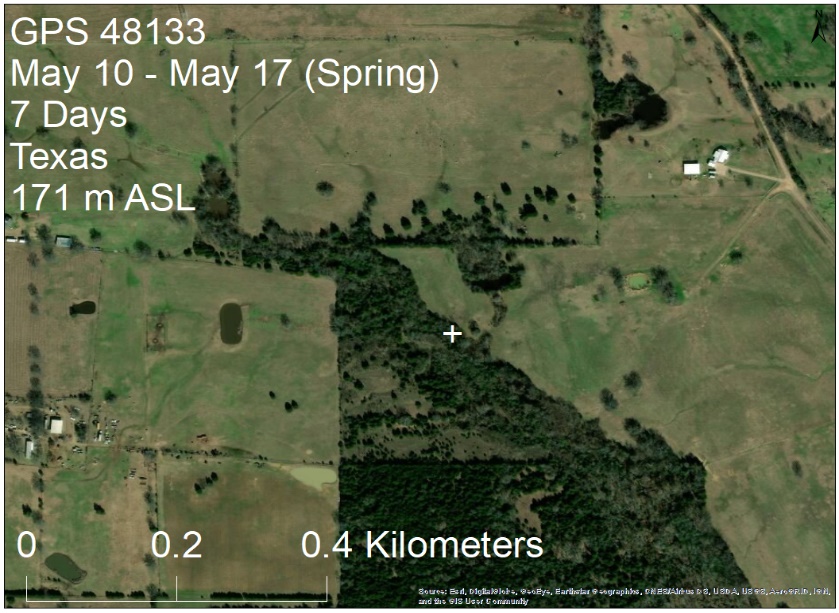 |
| --- | --- |

**Overwintering sites:**

| 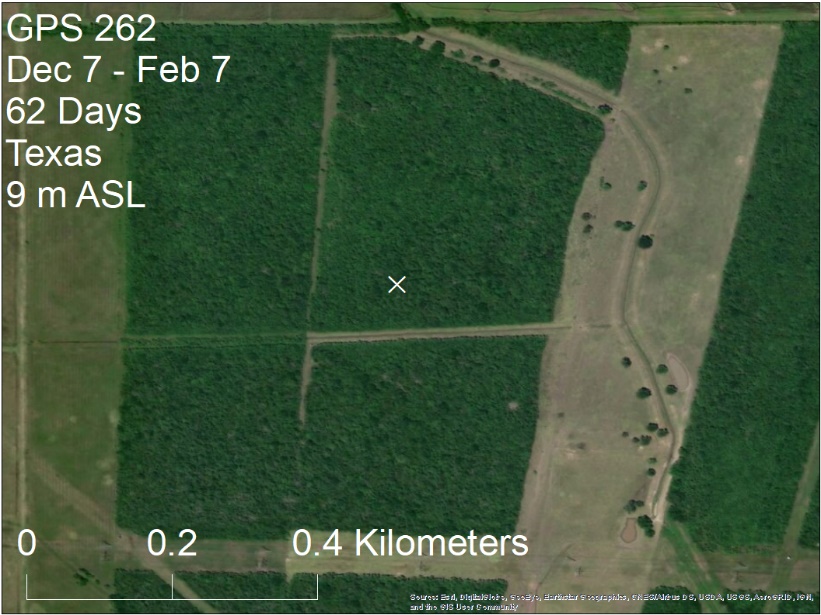 | 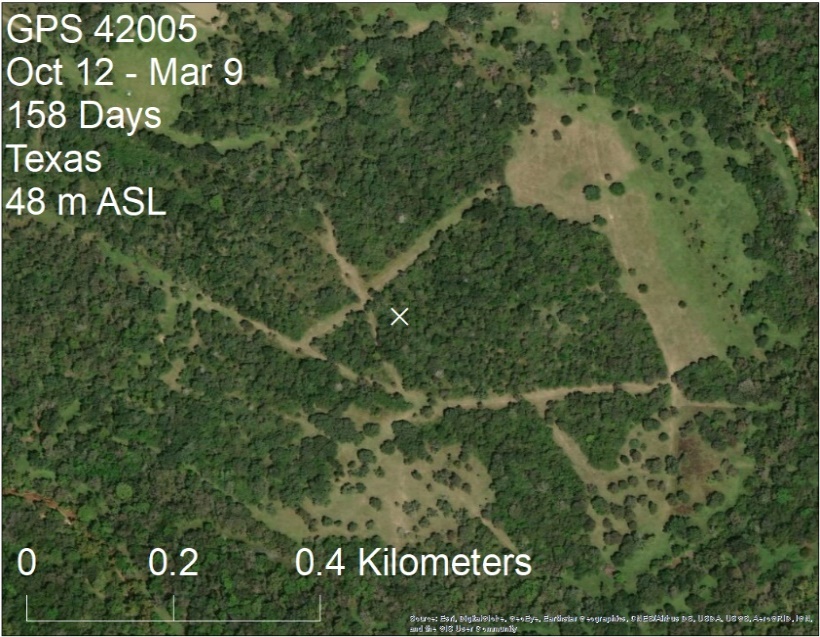 |
| --- | --- |
| 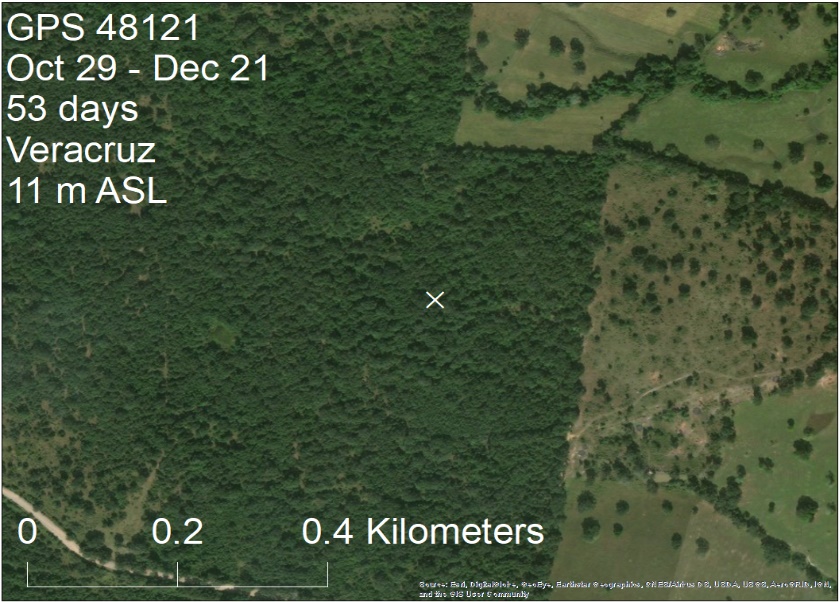 | 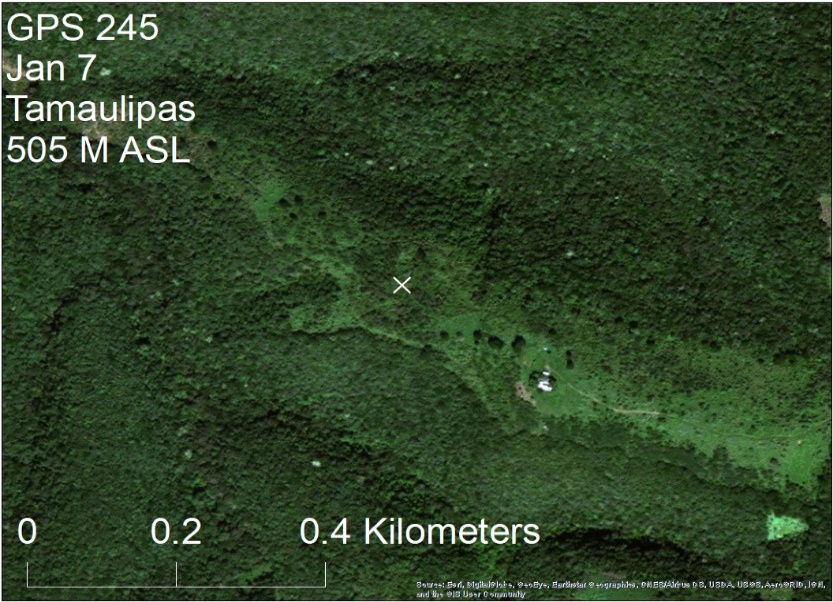 |
| 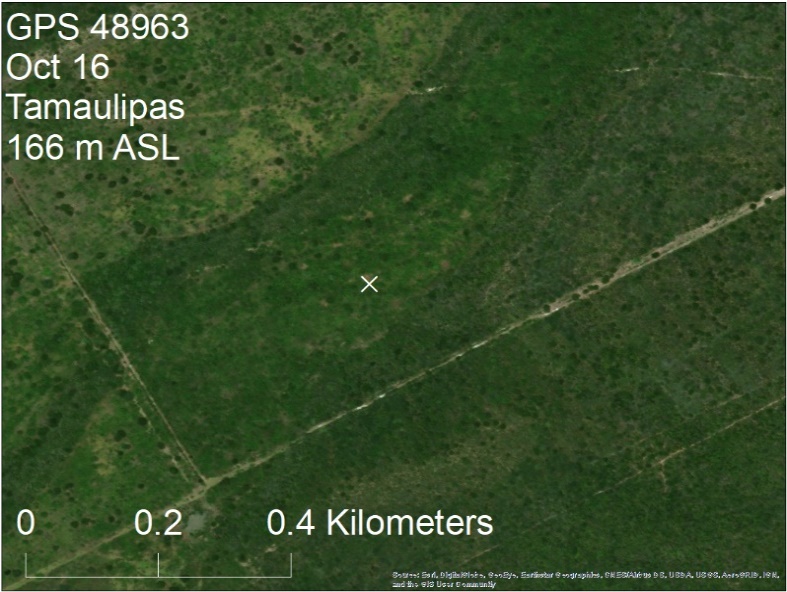 | 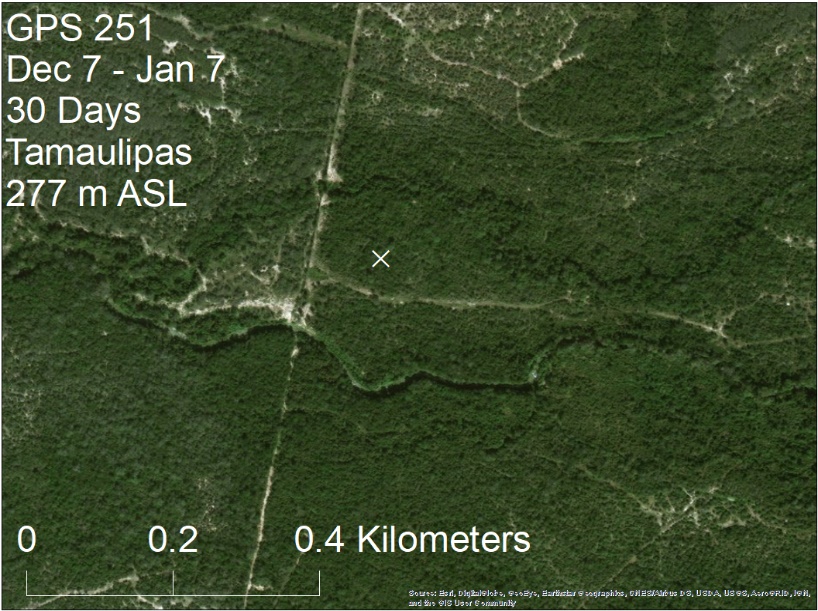 |
| 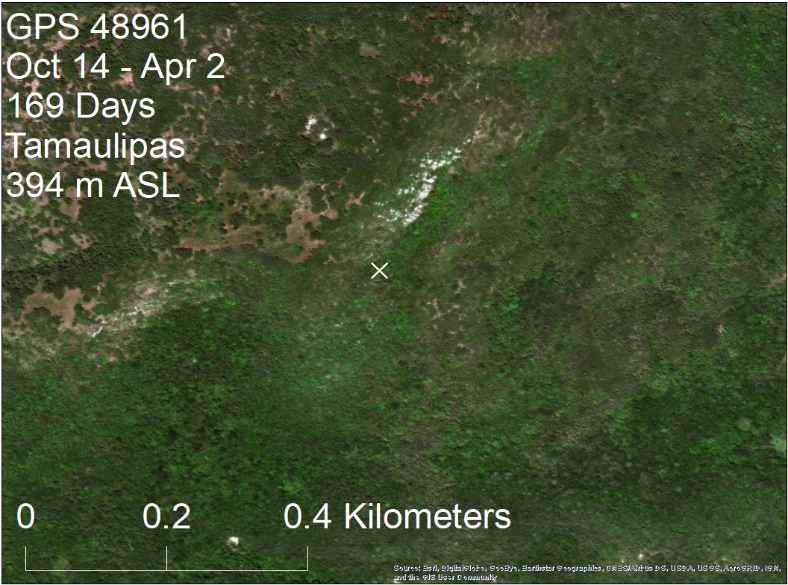 | 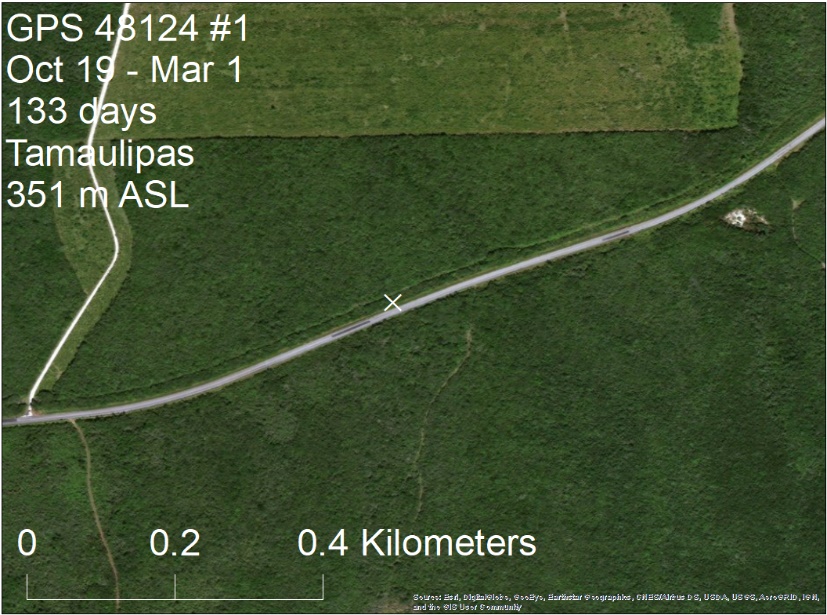 |
| 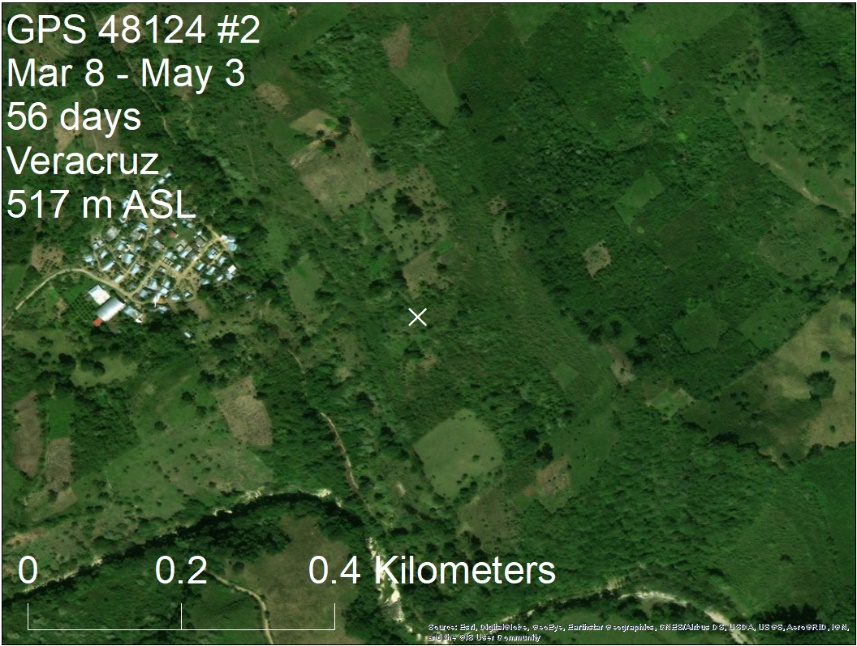 | 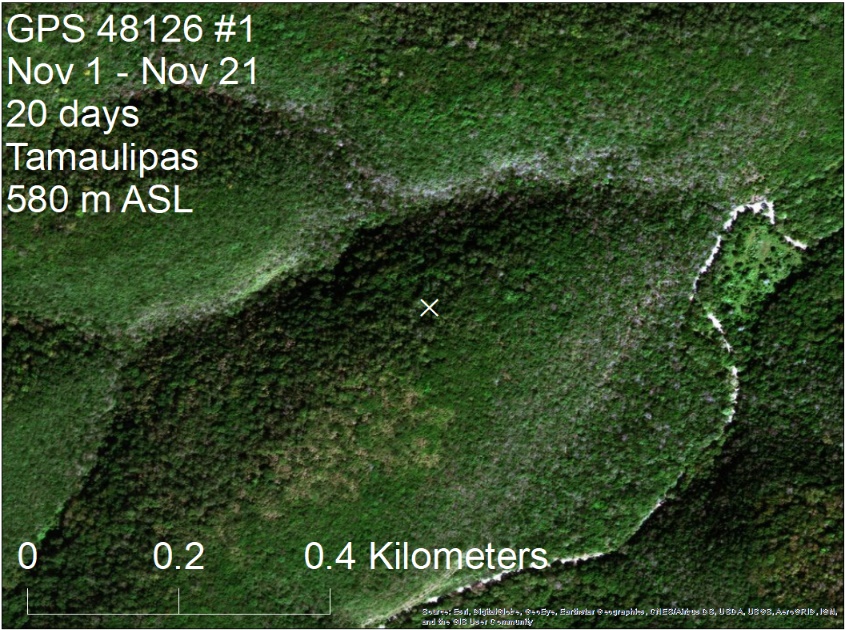 |
| 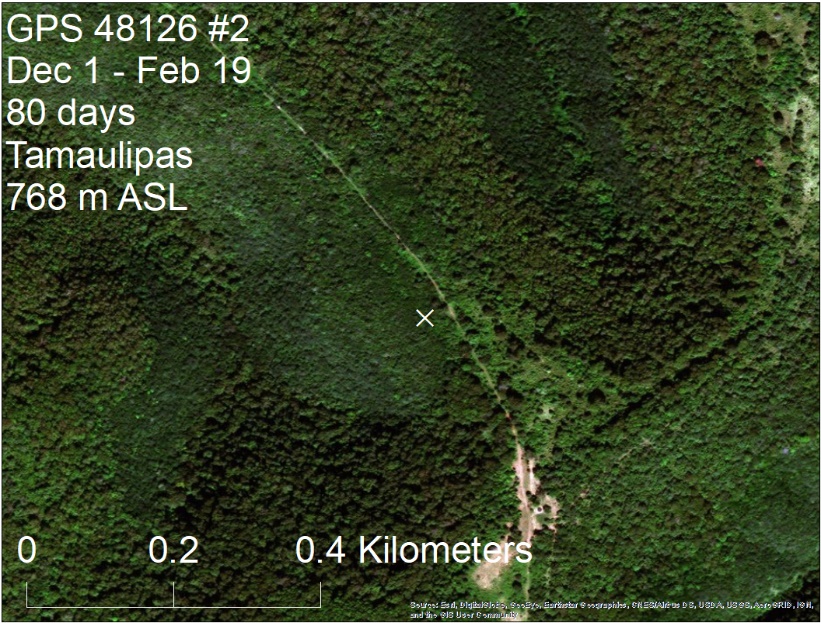 | 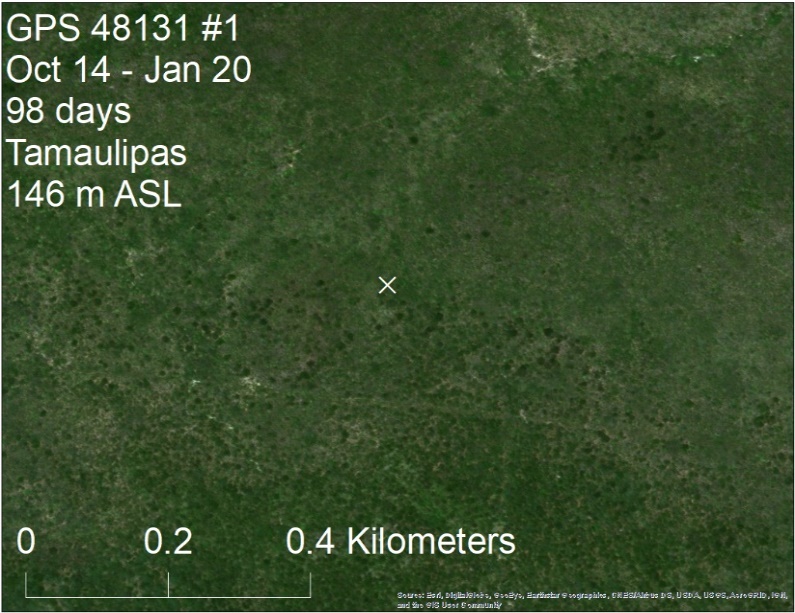 |
| 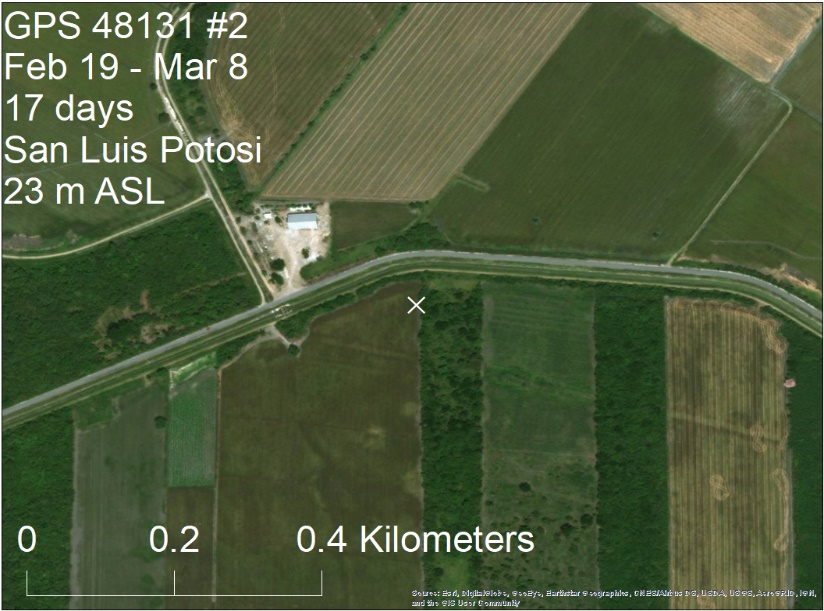 | 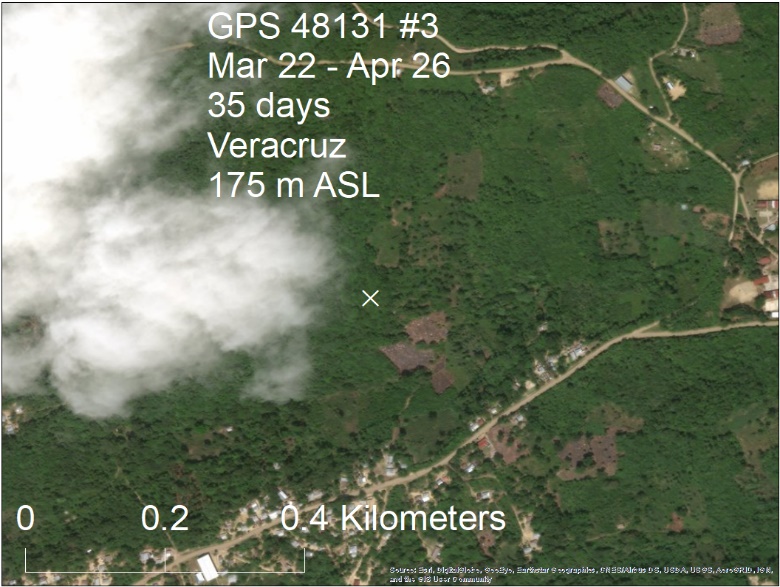 |
| 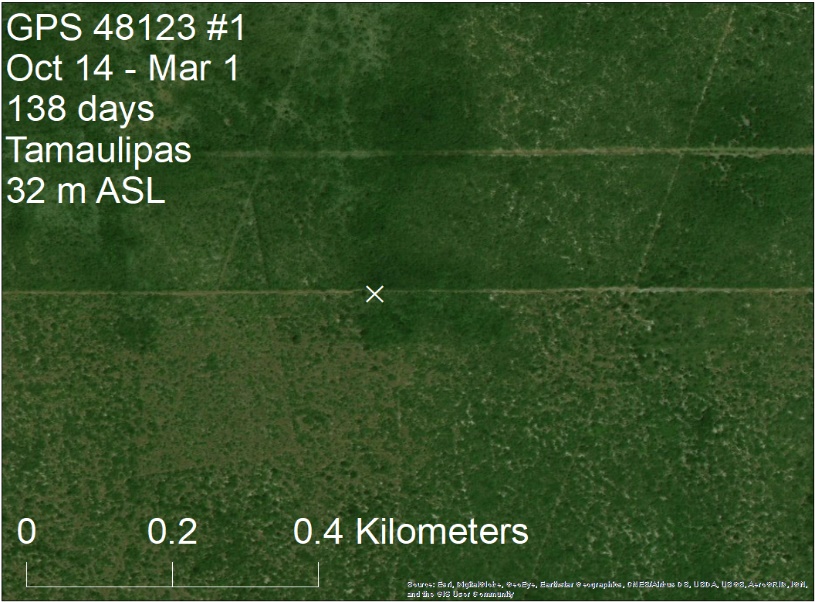 | 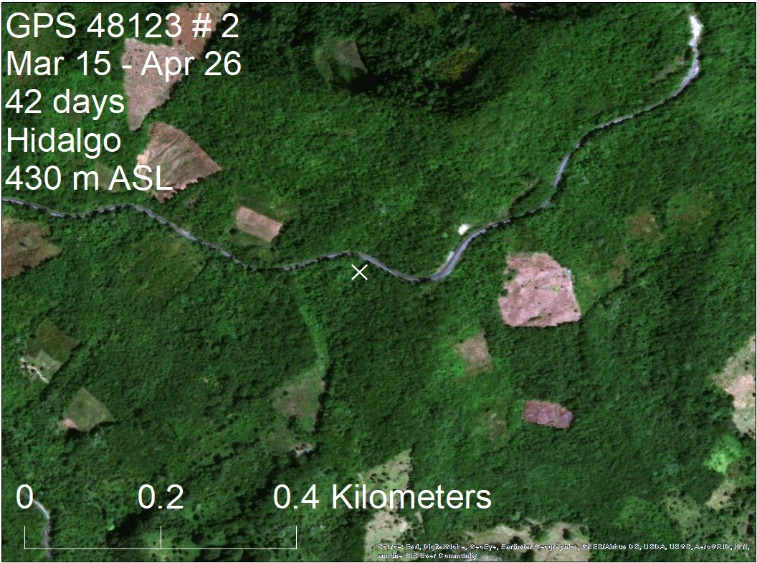 |
| 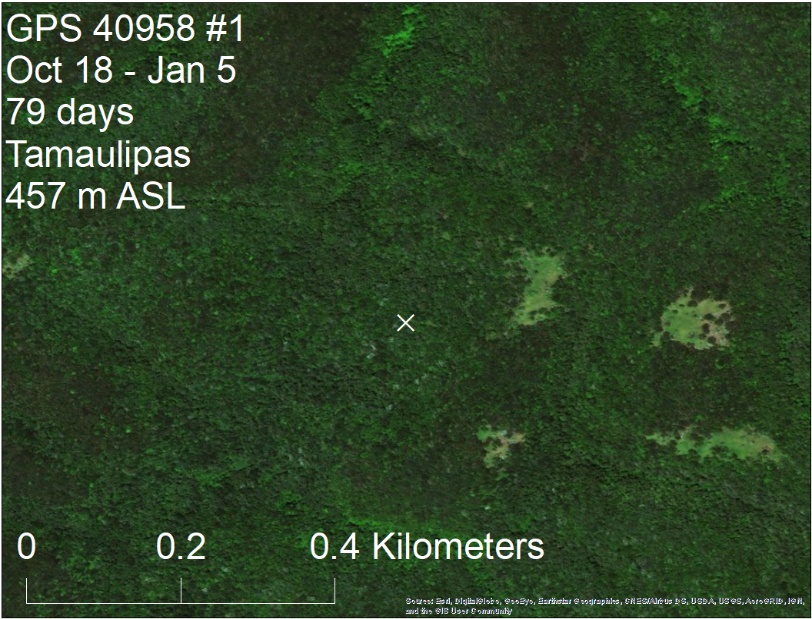 | 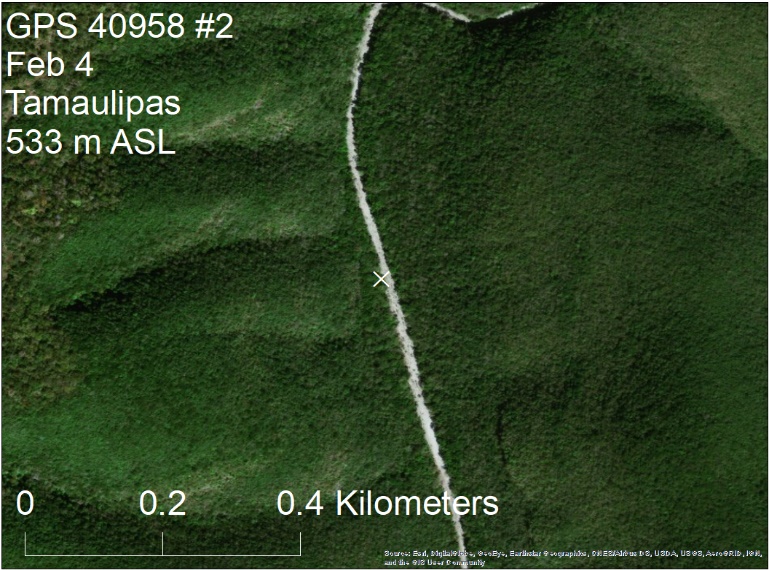 |
| 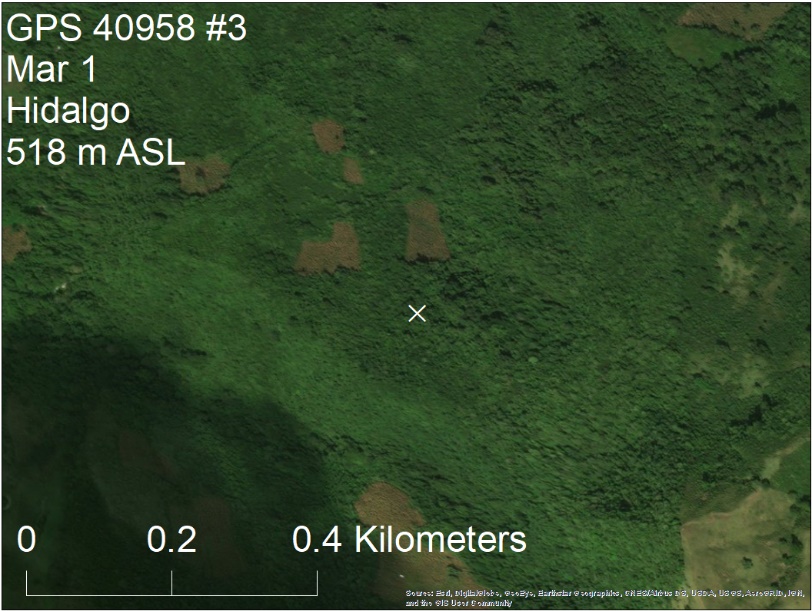 | 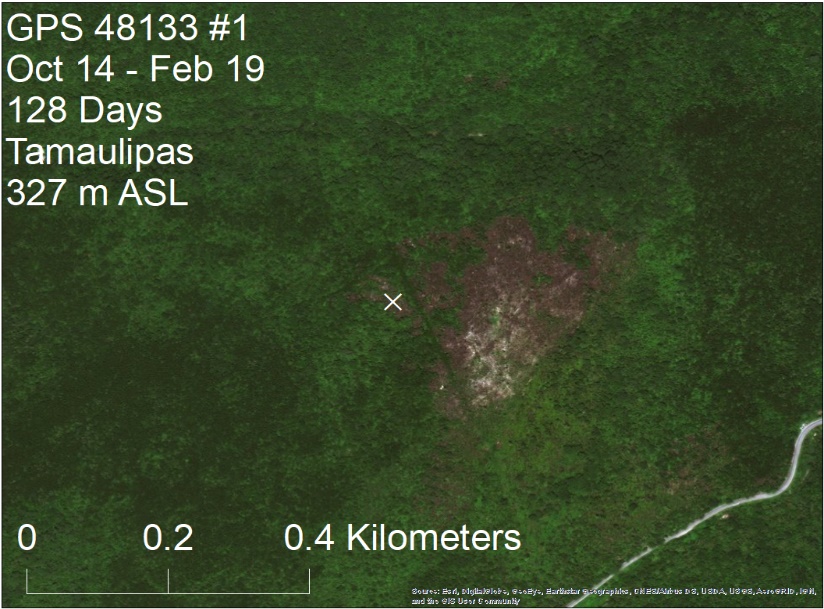 |
| 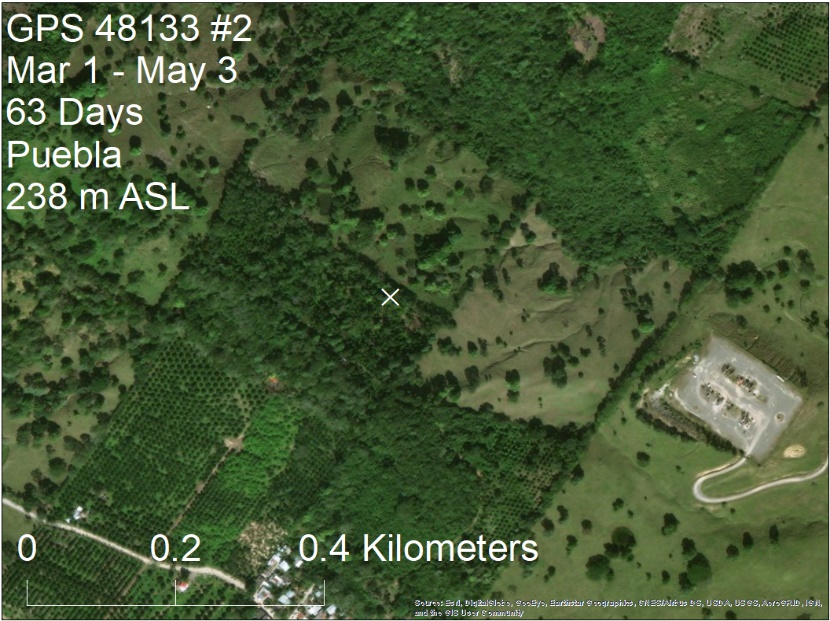 | 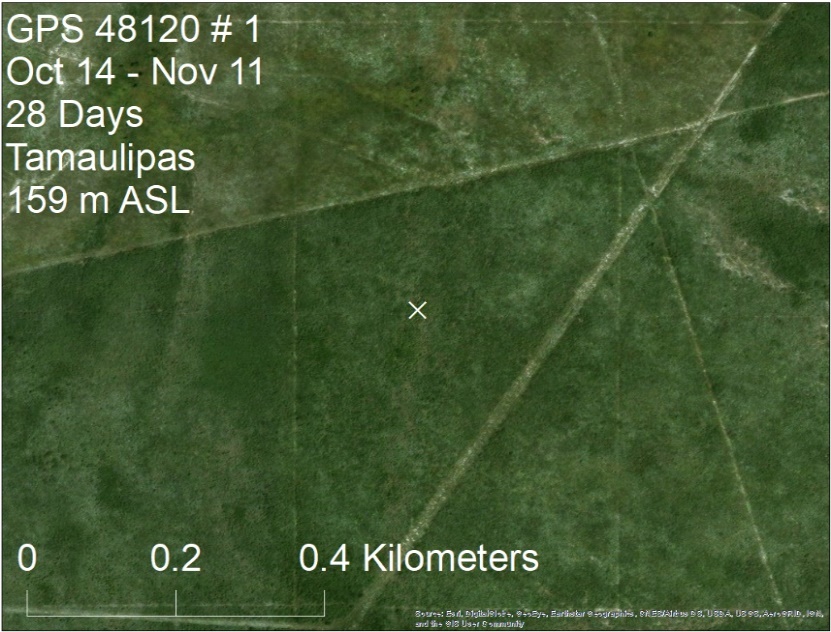 |
| 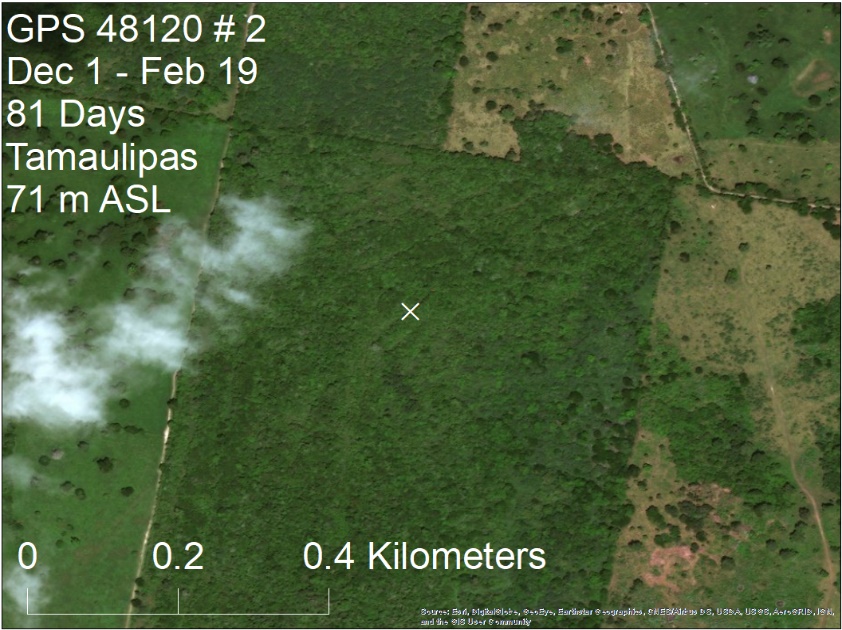 | 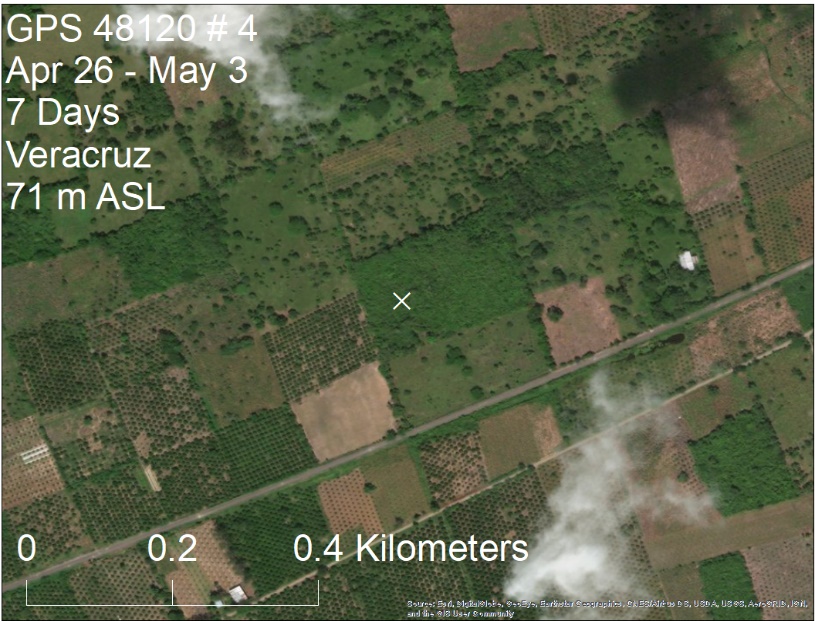 |
| 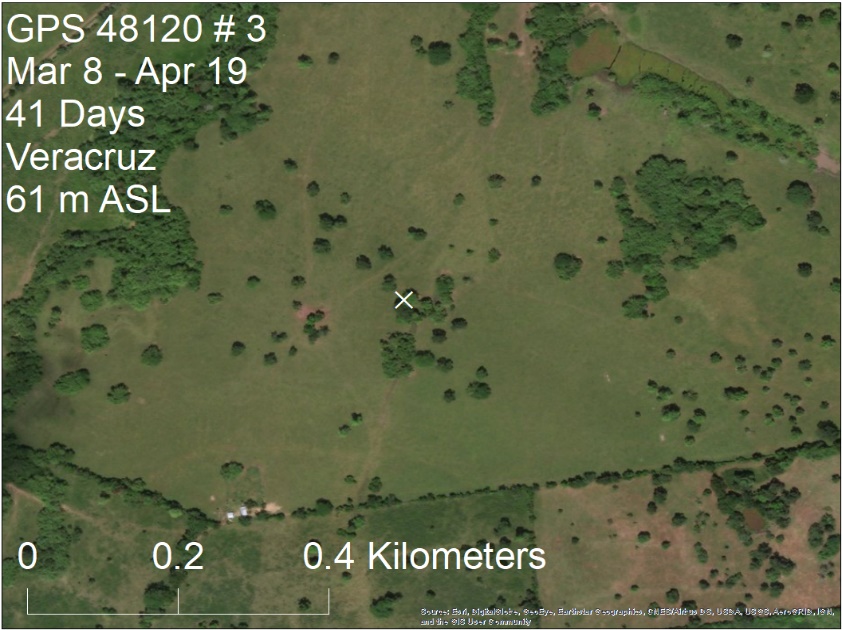 | 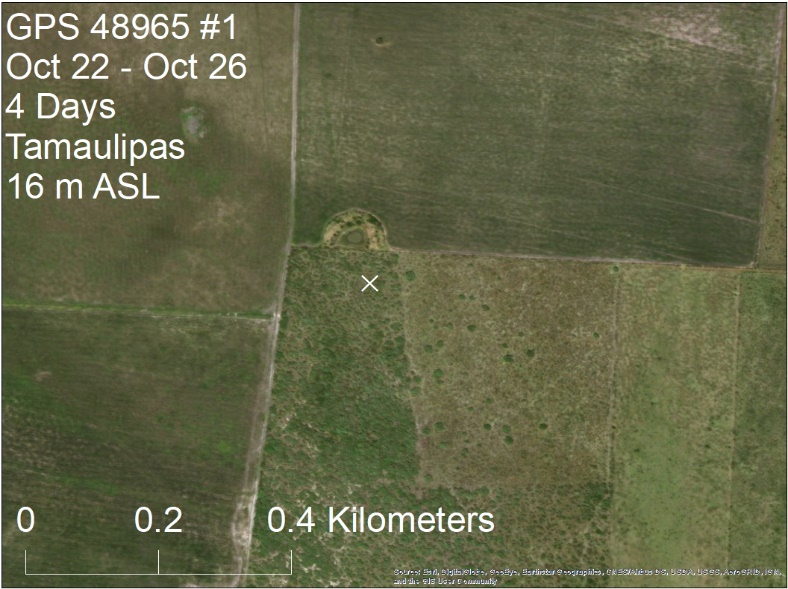 |
| 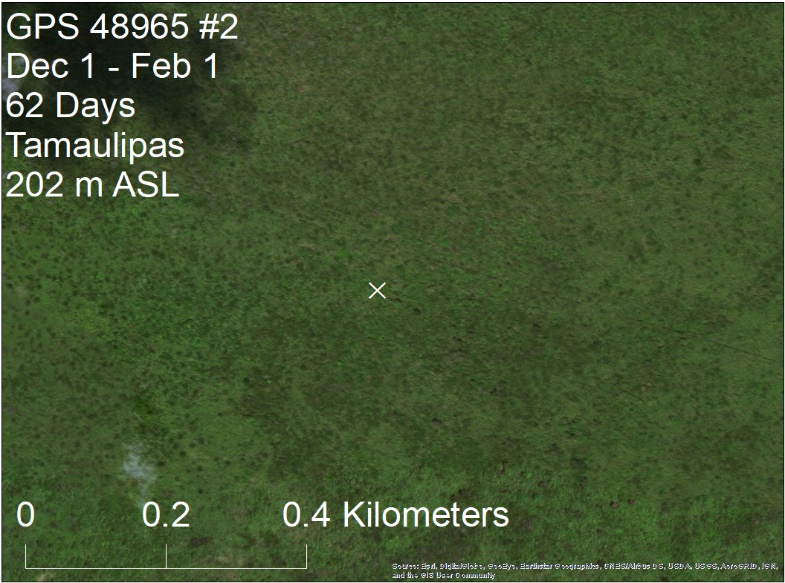 | 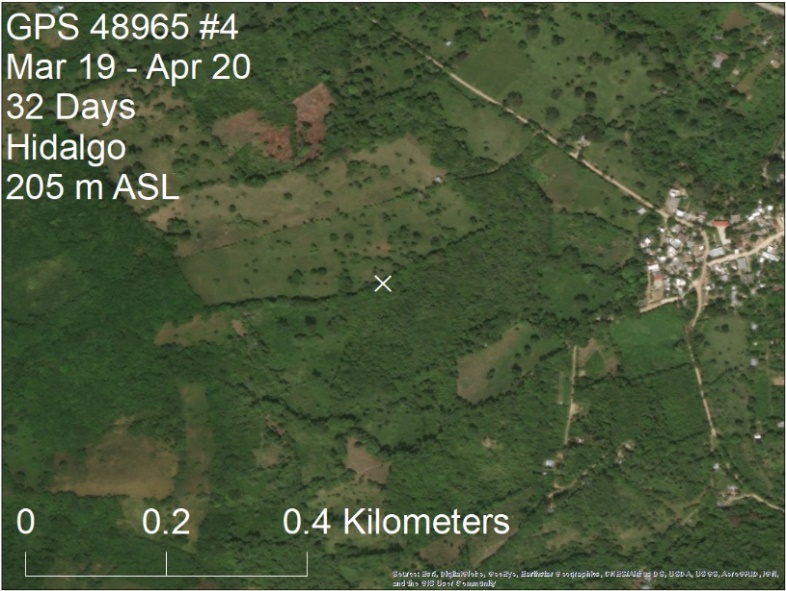 |
| 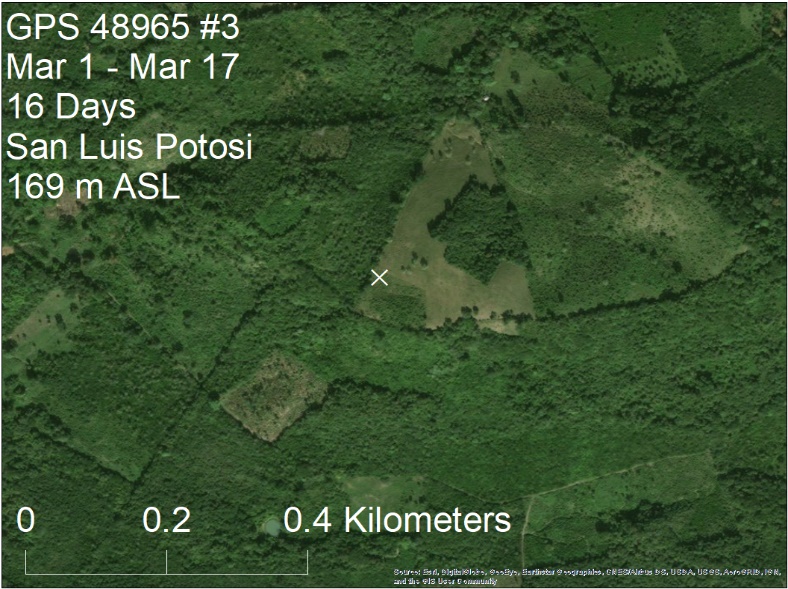 | 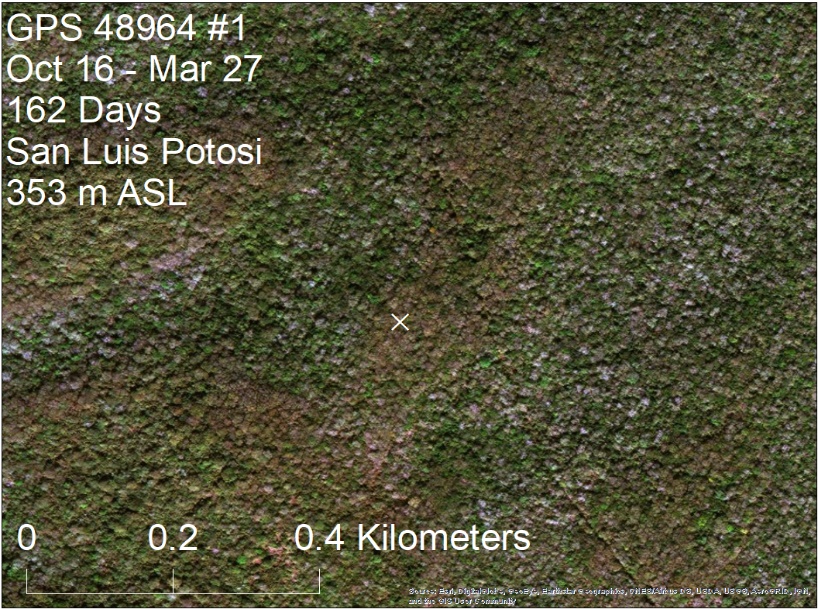 |
| 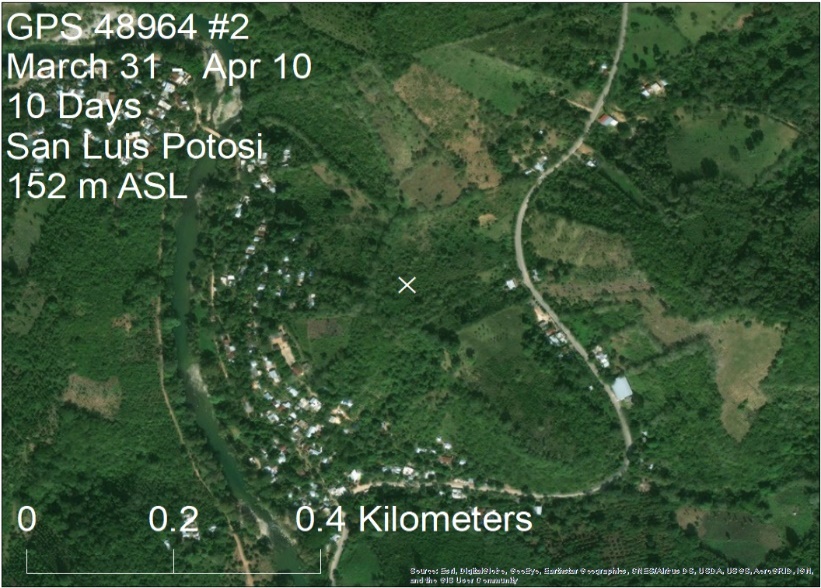 | 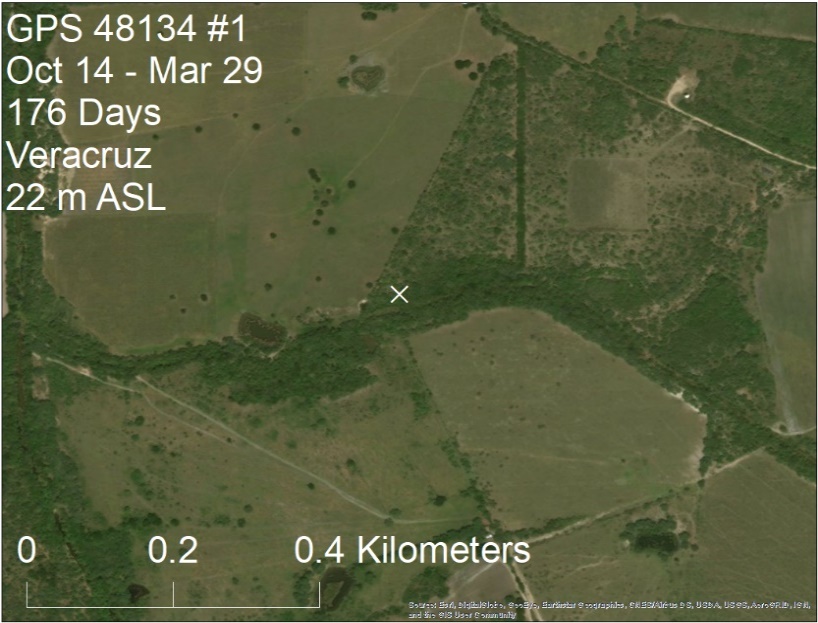 |
| 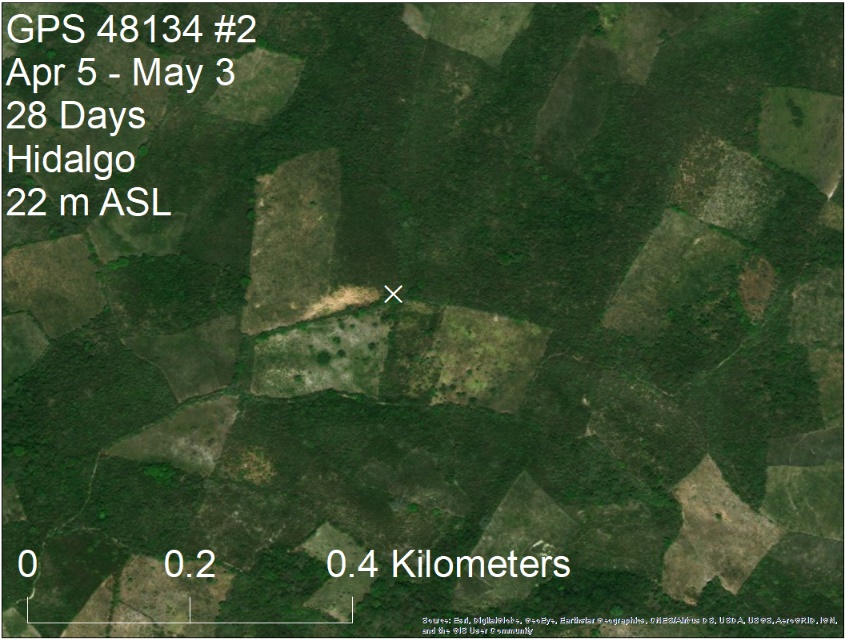 |  |
